# Primordial germ cell specification and early developmental cell states in Pacific oyster

**DOI:** 10.1186/s12864-025-12122-7

**Published:** 2025-10-23

**Authors:** Mackenzie R. Gavery, Lauren E. Vandepas, Lauren M. Saunders, Brent Vadopalas, J. Adam Luckenbach, Cole Trapnell, Steven Roberts

**Affiliations:** 1 Environmental and Fisheries Sciences Division, Northwest Fisheries Science Center, National Marine Fisheries Service, National Oceanic and Atmospheric Administration, 2725 Montlake Blvd E, Seattle, WA 98112 USA; 2 School of Aquatic and Fisheries Sciences, University of Washington, 3707 Brooklyn Ave NE, Seattle, WA 98105 USA; 3 Department of Genome Sciences, University of Washington, Seattle, WA USA; 4 Present Address: Centre for Organismal Studies, Heidelberg University, Heidelberg, Germany

**Keywords:** Primordial germ cell, Oyster, Embryogenesis, Single-cell RNAsequencing, Aquaculture, Gastrula

## Abstract

**Background:**

Primordial germ cells (PGCs) are the precursor cells of gametes and pivotal in understanding reproductive and developmental biology. Importantly, having a thorough understanding of PGC specification is leading to critical advances in sterility induction in aquaculture species. In shellfish, however, the ability to develop these approaches is hampered by the lack of information available regarding germ cell specification. The goal of this study was to identify genes uniquely expressed in these earliest germ cells of the economically and ecologically important bivalve mollusc, the Pacific oyster (*Crassostrea* (*Magallana*) *gigas*).

**Results:**

To capture specification of the PGCs - which represent a rare cell type - during embryonic development, we analyzed single-cell transcriptomes during cleavage, blastula, and gastrulation stages of *C. gigas* development. We identified cells in gastrulae that likely represent developing, distinct larval tissue types and organs, including muscles and shell gland, as well as undifferentiated cells. Using expression of the germ cell marker gene *vasa*, we identified cells in blastulae that likely represent the developing germ cell lineage that had yet to fully differentiate and segregate from somatic cell types. However, by the gastrula stage, *vasa* expression was limited primarily to a single cluster of cells. Other genes uniquely expressed in these *vasa*-positive cells include those with functions in transcriptional repression, chromatin architecture, and DNA repair, suggesting these cells represent oyster PGCs. Interestingly, some genes with no known homologies are also uniquely expressed in this cluster, perhaps representing novel PGC-associated genes in bivalves.

**Conclusions:**

We identified a suite of candidate genes that can be explored for their role in oyster PGC specification and advance efforts to develop methods to achieve reproductive sterility via germ cell disruption in cultured shellfish. In addition, this effort produced a transcriptional atlas of early developmental cell states in bivalve embryos, providing a wealth of information on genes contributing to other important developmental processes, such as tissue differentiation and shell production. These data represent the earliest developmental stages examined via single-cell RNA sequencing in a lophotrochozoan.

**Supplementary Information:**

The online version contains supplementary material available at 10.1186/s12864-025-12122-7.

## Background

Primordial germ cells (PGCs) are specialized cells that give rise to self-renewing germinal stem cells (GSCs; germline), which subsequently differentiate into gametes in the gonad. Because disruption of germline development can result in sterility, perturbation of PGC development is being explored as a means to produce sterile animals for use in aquaculture [1–4]. Sterile or non-reproductive shellfish are both a market-driven need and an ecologically sustainable approach to increasing food production via aquaculture. Sterility has clear advantages in shellfish aquaculture including the ability to improve rates of growth and meat quality, prevent accidental establishment of non-native species into the environment, and preclude genetic contamination of wild, native bivalve populations by farmed conspecifics [5, 6].

The Pacific oyster *Crassostrea* (*Magallana*) *gigas* ([7]; but see [8, 9]) is one of the world’s most economically important bivalves, approaching a million metric tons farmed annually [10]. *Crassostrea* sp. are also among the best characterized molluscan model systems for evolution and development (evo-devo), genetics, and cell biology (reviewed in [11]) and provide important ecosystem services [12]. Efforts to generate sterile oysters for aquaculture to date have involved creation of triploid (3n) individuals [5, 13, 14]. The shellfish aquaculture industry produces triploid oysters due to their reduced gonad development, year-round marketability, and improved performance traits when farmed [15–19]. Although use of triploids in aquaculture has been widely adopted, increasing reports of triploid mortality under environmentally stressful conditions [20–22] highlight the critical need for alternative methods to induce sterility in farmed oysters.

An alternative approach to achieving sterility in bivalves, that has the potential to avoid production issues associated with triploidy, is the induction of sterility via perturbation of genes essential for PGC formation. The power of this biotechnological approach has been realized recently in finfish species, where suppression of the germ-cell specific gene, *dead end* (*dnd*), produced several fish species with no detectable germ cells [1, 23, 24]. However, gaining a thorough understanding of the genetic programs underlying germ cell fate is a critical step toward controlling reproduction through molecular approaches such as these. In bivalves, PGC development is largely uncharacterized beyond descriptions of expression of the conserved germline marker gene *vasa* [25–28], a deadbox RNA helicase [29–31]. While other known marker genes for vertebrate PGCs and germline such as *dnd* [32] are not present in molluscs, previous studies in *C. gigas* have demonstrated that *vasa* is highly expressed in PGCs and necessary for normal oyster PGC development [25–27]. Gene expression analysis in early germ cells is challenging to assess because the germline is sequestered from somatic cells early in embryonic development in most animals (reviewed in [33]) and typically has relatively low amounts of transcriptional activity to protect it from introduction of mutations [34]. Identification of genes involved in oyster PGC specification has been elusive due to a lack of targeted sequencing efforts with sufficient cell-level resolution, as germ cell precursors make up only a small fraction of the cells in developing embryos.

Single-cell RNA-seq (scRNA-seq) has emerged as a powerful technique to identify and molecularly characterize rare cell populations in developing organisms, including other marine invertebrates [35–38], revealing transcriptional signatures associated with early specification and differentiation of cell types including of PGCs [35, 39–41]. In invertebrate gastrulae, scRNA-seq has been an effective technique to identify cell-type differentiation trajectories, discover novel genes associated with developing tissues, and better understand gene regulatory networks [35, 36, 42, 43]. While there are single-cell transcriptomic resources for oyster trochophore larvae [38] and a variety of adult tissues [44, 45], earlier developmental time points have not previously been reported in oysters or other lophotrochozoan taxa.

Here, we apply scRNA-seq to profile *C. gigas* early cleavage stages, blastulae, and gastrulae to comprehensively identify and characterize single-cell gene expression in whole developing animals with a primary focus on PGC specification. We show that PGC sequestration occurs prior to gastrulation and identify novel PGC-specific genes that will be used as candidates for future gene knockdown/silencing experiments to develop sterile shellfish for aquaculture efforts. We present the earliest single cell developmental time series atlas for a lophotrochozoan, data which provide novel insights into formation of a variety of larval tissues in the model bivalve *C. gigas*. We posit that these datasets are robust resources for exploring transcriptional activities, cell states, and cell-type differentiation early in oyster embryonic development.

## Methods

### Experimental design and C. gigas sampling

Gravid adult *C. gigas* were generously provided by Taylor Shellfish Hatchery (Quilcene, WA, USA). All steps were conducted at room temperature (approximately 20℃). Male or female gonad tissues were submerged in filtered seawater (FSW) (0.2 μm filter, salinity ~ 29 ppt.) and gametes gently removed by agitating the tissue using a lab spatula. Isolated oocytes were passed through a 90 μm filter, then collected with a 20 μm filter to remove debris. Oocytes were then allowed to hydrate for 1 h in FSW. Oocytes were visually assessed with a microscope to ensure complete hydration prior to fertilization. Spermatozoa were separated from the gonad of a male oyster using a lab spatula in approximately 20 mL of FSW. Sperm were observed with a microscope to qualitatively confirm motility. A small volume of sperm suspension was mixed with eggs to initiate fertilization (at 1:200 sperm solution to eggs). Mixed gametes were incubated for 10 min at room temperature. Fertilized oocytes were again screened on a 20 µM filter and rinsed with FSW to remove excess spermatozoa, and subsequently maintained in FSW during development.

**Fig. 1:**
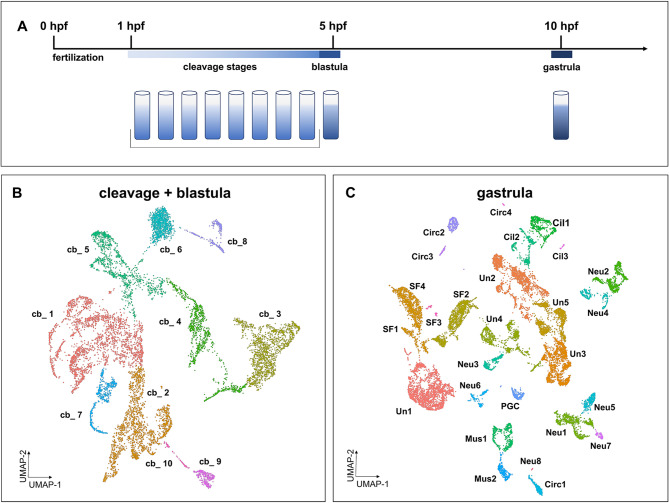
Single-cell transcriptional heterogeneity of early *C. gigas* embryos increases with developmental time. (**A**) Sampling design: cleavage-stage embryos were pooled into a single sample representing all cleavage stages. A sample of blastula-stage embryos were collected and sequenced in a separate library to allow the identification of the developmentally oldest cells in the pool. Gastrula-stage embryos sampled at 10 hpf for reference. (**B**) UMAP visualization of single-cell clustering of the cleavage (pre-blastula) + blastula stage (cb) that revealed 10 putative clusters. (**C**) UMAP visualization of single-cell clustering of the gastrula stage. Twenty-seven putative clusters representing broad cell types were resolved using UMAP clustering analysis (Cil, ciliated cells; Circ, circulatory system-related; Mes, undefined mesodermal derivatives; Mus, muscle-related; Neu, neuronal; PGC, primordial germ cell; SF, shell field; Un, undefined)

To measure genes uniquely expressed in *C. gigas* PGCs and/or PGC precursors, we collected embryos at the earliest reported developmental stages for the expression of *vasa* [26]. We collected early embryos from 8 time-points representing a continuum of development from cleavage- to blastula-stage embryos (Fig. 1A). Development in oysters can be asynchronous and samples were visually assessed to ensure that the majority (>50%) of embryos were at similar developmental stages. To collect cleavage-stage embryos, samples were taken after each cell division (~ 35 min) and placed on ice to arrest further development. Embryos were counted and 10,000 embryos for each of the first four cleavage samples, and 100,000 embryos for the remaining four cleavage samples (to account for the smaller proportion of cells that may represent the putative germ cells in the later cleavage-stage embryos) were pooled for cell dissociation and library preparation. To collect blastula-stage embryos, a sample was taken approximately 35 min after the last cleavage stage (approximately 6.5 h-post fertilization (hpf)). Approximately 300,000 blastula-stage embryos were collected in FSW for cell dissociation and library preparation. To collect gastrulae, embryos were sampled 10 hpf [46]. Approximately 40,000 gastrulae were collected in FSW for cell dissociation and library preparation.

### Cell dissociation and scRNA-seq library preparation

Gastrula-stage embryos were rinsed three times, while gradually decreasing the salinity: first in FSW, next in 0.75x calcium- and magnesium-free artificial seawater, salinity ~ 30 ppt (ASW), then in 0.5x ASW, and then finally in 0.3x ASW. Embryos were dissociated to single-cell suspensions with 500 µL of 10 mg/mL protease (Native *Bacillus licheniformis* protease; Creative Enzymes, Catalog Number: NATE0633) in 0.3x ASW incubated at room temperature for 15 min with intermittent gentle pipetting with a P1000 pipette. Cells were visualized on a microscope to monitor dissociation efficiency. The dissociation reaction was halted by transferring cells to ice-cold Dulbecco’s phosphate-buffered saline (dPBS, ThermoFisher; Catalog Number: 14190144, pH 7.0-7.3) containing 10% fetal bovine serum (FBS; Thermo Fisher; Catalog Number A4736401). The dissociated cells were filtered through a 30 μm filter into a 15 mL tube, centrifuged (600 rcf, 5 min at room temperature) and resuspended in 5 mL 0.3x ASW. This rinse was repeated, followed by resuspension in 200 µL 0.3x ASW. Cells were counted and viability assessed using 0.2% Trypan blue (HyClone; Catalog Number: SV30084) on a Bright-Line Hemacytometer (Hausser Scientific, Catalog Number: 3110). To process cleavage-stage and blastula samples, cell dissociation was performed similarly with the exception that embryos were rinsed once in room temperature FSW and then resuspended directly in dPBS for subsequent dissociation, filtering, and rinsing steps.

Cells were prepared for sequencing using the 10X Genomics Chromium platform [47]. Single-cell mRNA libraries were prepared using the Chromium Next GEM Single Cell 3’ GEM, Library & Gel Bead Kit v3.1 (10x Genomics, Pleasanton, CA, USA). Approximately 24,000 cells (split equally across 4 reactions) were targeted for the gastrula sample, approximately 18,000 cells (split equally across 3 reactions) were targeted for capture for the cleavage pool and 6,000 cells were targeted for the blastula sample. Quality control and quantification assays were performed using a Qubit fluorometer (Thermo Fisher, Catalog Number: Q33238) and a D1000 Screentape Assay (Agilent, Catalog Number:5067–5582). Libraries were sequenced on two Illumina NextSeq 500 75-cycle, high output kits (v2.5; Illumina, Catalog Number: 20024906).

### Processing of scRNA-seq reads

Sequencing reads were processed using the Cell Ranger pipeline (v3.1.0, 10X Genomics). A custom *C. gigas* STAR genome index was built using gene annotations from the “cggigas_uk_roslin_v1” version of the genome (GenBank: GCA_902806645.1) and filtered for protein-coding genes using the *mkref* command. Cell barcodes and unique molecular identifiers (UMIs) were determined using the Cell Ranger *count* command. To ensure accurate calling of cells, a more stringent filter requiring 2000 UMIs per cell was applied to cell calling from Cell Ranger (Fig. S1). GitHub repo with code: https://github.com/RobertsLab/pgc-edc-oyster.

In total, approximately 8 × 10^8^ GB of sequencing data were generated across the cleavage-stage, blastulae and gastrulae libraries (NCBI SRA: PRJNA906172). Post-filtering, a total of 18,511 cells (9,314 median UMIs and a median 2,828 genes per cell) were analyzed for the gastrulae, 7,658 cells (4,056 median UMIs and 1,949 median genes per cell) for the cleavage-stage embryos, and 2,836 cells (5,232 median UMIs and 2,277 median genes per cell) for the blastulae embryos. Mapping was consistent across libraries with 80–85% of reads mapping to the *C. gigas* genome. See Table S1 for additional sequencing and mapping information. Data for cleavage-stage and blastula cells were combined for downstream analysis.

### Dimensionality reduction, clustering and cell-type annotation

After cell calling and quality control, single cell data analysis was performed using Monocle3 [48] (v1.3.4), following a standard processing pipeline: (1) log normalization of gene counts and PCA analysis with retention of the 20 top principal components for (2) projection of data into two dimensions with Uniform Manifold Approximation and Projection (UMAP) [49] (*umap.min_dist = 0.2*,* umap.n_neighbors = 15 L*) and (3) Leiden clustering to group cells. UMAP visualization and clustering was performed separately for the gastrulae, and combined for the cleavage-stage and blastula embryos. The function *align_cds* [50] was used to minimize batch effects of replicate libraries in low dimensional space. All genes were provided as input into Principal Component Analysis (PCA). For cell clustering, we manually adjusted the resolution parameter towards modest overclustering. Genes that were highly expressed and specific to a particular cluster (referred to here as ‘marker genes’) were identified in order to facilitate the annotation of cell clusters to a particular cell type. Specifically, the function *top_markers (group_cells_by = “cluster”*, *reference cells = 1000* (Monocle3 v.1.3.4 [47, 51] was used to identify the genes most specifically expressed in each cluster. The 25 most specifically expressed genes by “marker_score” (a value between 0 and 1 based on the fraction of cells expressing the gene scaled by a measure of how specific the gene’s expression is to the cluster) are reported in Tables S2-S4.

### Annotation of C. gigas genes to homologs in model invertebrates

In order to facilitate direct comparisons of *C. gigas* marker genes identified here to those in other model marine invertebrates, an iterative BLAST was performed to *Caenorhabditis elegans*,* Drosophila melanogaster* and *Strongylocentrotus purpuratus* databases (the top BLAST hit and corresponding e-value reported in Tables S2-S4). Specifically, the full set of *C. gigas* genes (GCF_902806645.1_cgigas_uk_roslin_v1_translated_cds.faa) was compared to SwissProt database (20210613_ncbi_sp_v5/swissprot (blastp)), *C. elegans* protein database (Caenorhabditis_elegans.WBcel235.pep (blastp)), *D. melanogaster* nucleotide database (dmel-all-CDS-r6.37 (tblastn)) and the *S. purpuratus* protein database (ProteinsSpur5.0 (blastp)) using NCBI BLAST [52]. Marker genes used to assign cell or tissue identities to UMAP clusters were manually screened using iterative reciprocal protein BLAST to assess predicted gene homology.

### Semi-quantitative PCR

RNA was extracted from pooled embryos (6 hpf blastula) or larvae (18 hpf trochophore, 26 hpf D-hinge) and whole tissue from 8-month-old juveniles using Tri Reagent (ThermoFisher, Catalog Number: 15596018), following the manufacturer’s protocol. The amount of cDNA was normalized and 200 ng of cDNA were loaded into each PCR reaction. PCR amplifications were performed in a 25 mL reaction volume consisting of 9.5 µL nuclease-free water, 12.5 µL GoTaq master mix (Promega, Catalog Number: M7122), 1 µL 10 µM forward primer, 1 µL 10 µM reverse primer, 1 µL cDNA template. Amplification was performed on a T100 Thermal Cycler (Bio-Rad): initial denaturation, 2 m at 95 °C, followed by 35 cycles of 30 s at 95 °C for denaturation, 30 s at 60 °C annealing, 60 s at 72 °C for extension, and final extension at 72 °C for 10 min. The PCR products were then assessed by gel electrophoresis at 100 V for 30 min in a 1.25% (w/v) agarose gel in 1 x TAE buffer using SYBR Safe (Thermo Fisher, Catalog Number: S33102).

*GAPDH* was used as a housekeeping control gene and expression of PGC-related genes *nanos*,* vasa*,* sperm-specific protein PHI-2B/PHI-3* (*spPHI*), *uncharacterized gene LOC105328839* were assessed using semi-quantitative PCR (Table S5). Two biological replicates (either pooled larvae from different parent crosses or individual juvenile oysters) and at least two technical replicates were performed for each timepoint for each gene. Gel band quantification was performed using FIJI software [53].

### Hybridization chain reaction (HCR)

We performed HCR on oyster gastrula (10–14 hpf) following the cephalopod HCR protocol (HCR 3.0, Molecular Instruments, Los Angeles, CA, United States; [54, 55], with the following modifications. Samples were fixed in 4% paraformaldehyde in FSW overnight at 4 °C, washed thoroughly with PBS + 0.1% Tween-20 (PTw), and proceeded immediately without exposure to methanol. HCRs were performed in a 24-well flat-bottomed culture plate. Probes for oyster HCR were as follows: *shematrin* (LOC105342064; 20-probe set; Amplifier B4; Fluorophore 546), spPHI (LOC105327445; 20-probe set; Amplifier B3; Fluorophore 488), and *nanos* (LOC105347430; 20-probe set; Amplifier B2; Fluorophore 647). Following hairpin incubation, samples were washed 6 times in 5X SSC + 0.1% Tween-20, counterstained with DAPI, and imaged. Imaging was performed on a Nikon Ti (Eclipse) inverted microscope with Ultraview Spinning Disc (CSU-X1) confocal scanner (Perkin Elmer). Images were captured with an Orca-ER Camera using Volocity (Quorum technologies) and processed using FIJI [53].

## Results

We applied single-cell transcriptomics to analyze gene expression profiles in *C. gigas* early developmental stages: combined early cleavage-stages through blastulae and gastrulae (Fig. 1A). A total of 7,658 and 2,836 cells for the cleavage-stage and blastulae embryos respectively and 18,511 cells were analyzed for the gastrulae. Due to maternal mRNAs and undifferentiated cell states [56, 57], sequencing data from the earliest developmental stages (cleavage + blastula embryos) yielded few distinct cell clusters (Fig. 1B). In contrast, gastrula stage embryos showed a high degree of heterogeneity (Fig. 1C) and we identified early genes associated with tissue progenitors such as PGCs, shell field, muscle, and neurons (Table 1; Fig S2; Table S6).

**Table 1:** Gastrulae cluster identification.** ‘**Selected informative expressed genes’ were identified using a combination of highly conserved bilaterian marker genes, previously identified genes expressed in specific tissues or cells in other molluscs, and genes identified from the top marker analysis (see table S6 and Fig. S2 for gene identification numbers and visual representation of expression respectively)

UMAP Cluster	Cluster Name	Selected Informative Expressed Genes	Associated Germ Layer	Associated Tissue or Cell Type
1	Un1	HoxA7, HoxA1, HoxA5, HoxB7, eve, bmp1, bmp3, HTR	mesoderm	undefined
2	Un2	myosin ii HC, gsc, cdx, bmp2/4, gata2/3	endomesoderm	undefined
3	Un3	foxA, gsc, twist, chordin, dachshund, six3/6, bmp2/4, sox2, caveolin-3	ectomesoderm	undefined
4	SF1	HoxA5, keratin, shematrin, prisilkin-39, bmp1	ectoderm	shell field
5	Un5	brachyury, cdx, foxA	mesoderm?	gut-associated?
6	SF2	bmp1, bmp2/4, bmp3, chs2, keratin, shematrin, prisilkin-39, gsc, sox2, gata2/3, HoxA5	ectoderm	shell field
7	Un4	brachyury, cdx, snail2b, gata2/3, myosin LC, HoxB7, bmp3, sox2	ectomesoderm	undefined
8	Neu1	six3/6, TRPM2, FMRFaR, sox2, foxA	ectoderm	neural
9	Neu2	otp, gsc, HoxA1, bmp3, six3/6, prospero, sox2, FMRFaR, HTR, VMAT, four-jointed	ectoderm	neural
10	Cil1	zinc finger C2HC domain-containing protein 1 C, CFAP44, CROCC, DNAH7, CFAP77, four-jointed	ectoderm	ciliated cells
11	Mus1	bmp3, myosin LC, myosin HC, dachshund, brachyury, snail2a, snail2b, twist, otp	endomesoderm	muscle
12	Cil2	TUBA1A, RSPH1, HTR1, four-jointed	ectoderm	ciliated cells
13	Neu3	pax6, LHX5, bmp1, PCDH11, myosin HC II-like	ectoderm	neural, sensory
14	Neu4	snail2b, six3/6, neurogenin-1, HTR, four-jointed, sox11	ectoderm	neural
15	Neu5	six3/6, DOPA decarboxylase, HHIPL1, gat3, dachshund, SERT	ectoderm	neural
16	Circ1	dachshund, foxA, caudal, cripto	ectomesoderm	circulatory
17	Neu6	PRRX2, sup-9, homeobox unc-42, caudal, VMAT	ectomesoderm	neural
18	Mus2	dachshund, myosins, bmp3, caudal, sox11	endomesoderm	muscle
19	PGC	nanos, vasa	PGCs	germ cells
20	Circ2	fli-1, nkx2.5	mesoderm?	circulatory
21	Circ3	myophilin, ZIC-4, rax, SBSB3, FMRFaR, nkx2.5	mesoderm?	circulatory
22	Neu7	six3/6, dachshund, foxQ2, FMRFaR, SERT	ectoderm	neural
23	Cil3	myosin HC II/trichohylin, foxJ	ectoderm	ciliated cells
24	Cil4	HTR1	ectoderm	ciliated cells
25	SF3	sodium-dependent glucose transporter 1 A, mucin-17, HoxA5	ectoderm	shell field
26	SF4	shematrin, cytoskeletal keratins, HoxA5	ectoderm	shell field
27	Neu8	dachshund, DUXB, sox11, cdx, foxA	ectoderm	neural, sensory

### Early developmental origins of PGCs in the oyster C. gigas

#### PGCs are transcriptionally distinct by the gastrula stage in the oyster C. gigas

**Fig. 2:**
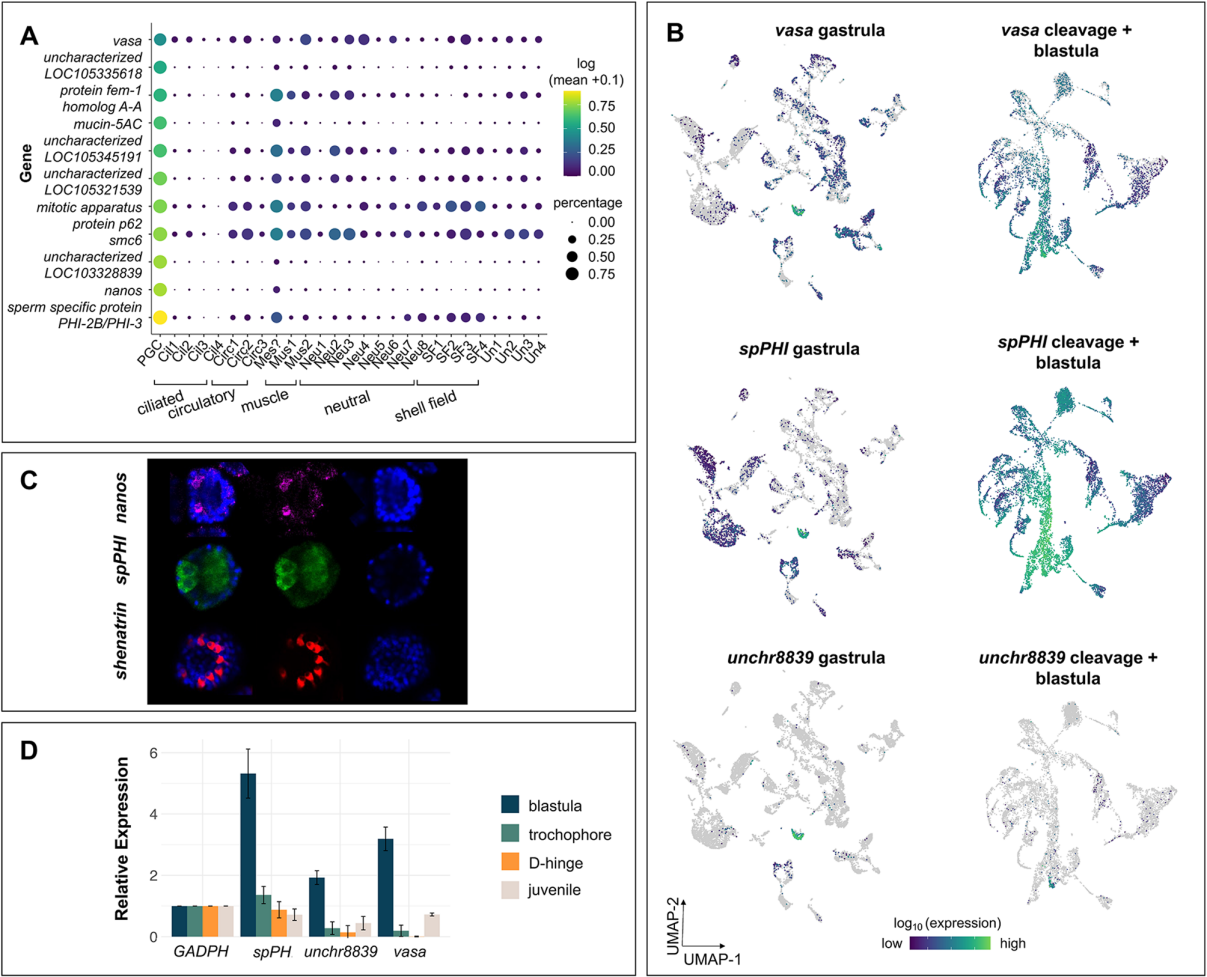
Tracing the expression of *vasa* identifies a single putative PGC cluster in *C. gigas* gastrulae. (**A**) Dot plot heat map displaying the expression of marker genes identified in the PGC cluster in gastrulae across all clusters. Dot color represents expression level and dot size represents fractional representation of cells expressing a gene. (**B**) UMAP visualization of expression of select PGC marker genes in gastrulae (left) and cleavage + blastula embryos (right): *vasa* expression is primarily limited to a single cluster in the gastrula stage whereas expression is broader in the earlier cleavage + blastula stages (top); *spPHI* shows high specificity to the PGC cluster in gastrulae, while expression is widespread in cleavage + blastulae embryos (middle); an uncharacterized oyster-specific gene (*LOC105328839*) shows high levels of expression specifically in PGC cluster in gastrulae, but is only expressed in a small number of cells in cleavage + blastula embryos (bottom). (**C**) HCR localization of expression of candidate PGC genes spPHI and nanos, and a shell field gene shematrin. Samples were counterstained with Hoechst. (**D**) Expression of selected genes that are highly expressed in the putative PGC cluster in gastrulae: spPHI (LOC105327445), uncharacterized protein (LOC105328839), and vasa (LOC105335166) across life history stages from whole tissues as determined by semi-quantitative PCR. Results were normalized to GAPDH (LOC105340512) expression

To identify PGCs in developing *C. gigas* embryos and determine co-expressed genes that may be important for oyster PGC specification we first searched our datasets for potential PGCs using the previously characterized oyster germline marker gene *vasa* (Fig. 2; Table 2) [25, 26]. In our *C. gigas* gastrulae dataset, one cell cluster showed both high expression of *vasa* and also had the highest proportion of *vasa*-expressing cells within the cluster (Fig. 2A-B). Although *vasa* expression was relatively specific to this cluster at this developmental stage and one of its top 25 marker genes, *nanos*, an evolutionarily conserved repressor of transcription shown to be expressed exclusively in the oyster germline [58, 59] showed even higher cluster specificity, providing further support that the cells in this cluster represent developing PGCs (Fig. 2A-B; Table 2). The specific expression of both *vasa* and *nanos* indicates that this single cluster of cells likely represents PGCs in oyster gastrulae (Fig. 2).

**Table 2:** Top 10 marker genes for the PGC cluster in *C. gigas* gastrulae

Gene ID	Gene Name	Marker Score	Swiss Prot ID	Conserved Domains	Protein IDs
LOC105328839	uncharacterized LOC105328839	0.73	N/A	N/A	XP_034312373.1
LOC105347430	protein nanos	0.70	P60321	zinc finger - Nanos superfamily (cl05351)	XP_011454832.3
LOC105327445	sperm-specific protein PHI-2B/PHI-3	0.63	Q3HNG7	linker histone 1/5 domain (cl00073)	XP_011426236.2
LOC105318512	mucin-5AC	0.63	N/A	N/A	XP_011413998.2
LOC105335618	uncharacterized LOC105335618	0.60	N/A	N/A	XP_034338648.1
LOC105321539	uncharacterized LOC105321539	0.51	N/A	N/A	XP_011418154.2
LOC105345191	uncharacterized LOC105345191	0.36	Q93075	metallo-dependent_hydrolases (cl00281)	XP_011451571.2
LOC105321610	structural maintenance of chromosomes protein 6	0.35	Q6P9I7	RecF/RecN/SMC N terminal domain (cl37666), Smc (cl34174)	XP_034314486.1
LOC105346676	mitotic apparatus protein p62	0.34	P91753	Nucleoplasmin-like domain (cl03870)	XP_034328868.1
LOC109619157	protein fem-1 homolog A-A	0.32	Q7T3P8	Ankyrin repeats (cl39094)	XP_034314173.1

Marker Score = the fraction of cells expressing the gene scaled by specificity

This cluster (which we will hereafter refer to as PGCs) also specifically expressed genes involved in transcriptional silencing, stemness, germline and gamete development, and chromatin remodeling and maintenance (Fig. 2A; Table 2). Many genes uniquely expressed in the oyster gastrula PGC cluster have previously been associated with germline development and maintenance in other animals, a pattern that has been described previously [58]. For example, the gene with the highest overall expression in the PGC cluster is a protamine-like histone variant, annotated as ‘sperm-specific protein PHI-2B/PHI-3’ (*spPHI*) (Fig. 2; Table 2). An orthologous gene has been described in the bivalve *Mytilus californianus*, where it is uniquely expressed in spermatozoa [60]. Interestingly, there are also genes uniquely expressed in the PGC cluster with unknown germline functions (Fig. 2A; Table 2). These include an uncharacterized *C. gigas* gene *LOC105328839* (Fig. 2A-B; Table 2), a mollusc-specific protein that exhibits some amino acid sequence homology to mucins.

We also performed spatial gene expression (HCR) in mid-to-late stage gastrulae to localize the expression of select PGC-associated genes (*nanos* and *spPHI*). To spatially orient the embryonic patterns of gene expression in gastrulae, we additionally performed HCR for the shell field gene *shematrin* (Fig. 2C). The *spPHI* gene, described here in oysters for the first time, is most highly expressed in PGCs in our gastrulae sequencing data and shows lower levels of expression in shell field and muscle clusters. HCR images of *spPHI* show several areas of expression, appearing as lobe-like circles on both regions of the embryos. We found that *nanos* has biased expression in one area of the embryo in two small groupings of cells, in agreement with previously reported expression data by Xu et al. [59] who observed two clusters of *nanos* expression in gastrula and later larval stages. *Shematrin* expression is localized to the developing dorsal side of the embryo, similar to patterns of shell field-associated genes described previously in oyster trochophores [38].

These data taken together support the identification of a specific cluster of cells in our single-cell sequencing analysis that likely represents PGCs in *C. gigas* gastrulae.

#### Some genes associated with PGCs are expressed prior to gastrulation

Cells from the earliest cleavage stages (2- to 512-cell) through the blastula stage were analyzed together to identify genes uniquely expressed in the earliest germ cells and to potentially track PGC formation in *C. gigas* (Figs. 1B and 2B). Genes associated with PGC development were expressed at these early developmental stages. Early embryos display wide expression of *vasa*; however, some clusters show relatively elevated levels of *vasa* expression (Fig. 2B). Many marker genes identified as being uniquely expressed in the gastrula PGC cluster were either not detected or showed broad expression in the cleavage-blastula cells (Fig. S3). Interestingly, *spPHI*, was co-expressed in *vasa*-expressing cells in these early embryos (Fig. 2B).

To temporally assess expression of selected PGC marker genes identified through our sequencing analysis, we performed semi-quantitative PCR on pooled blastulae, trochophores, and early D-hinge larvae, as well as individual 8-month-old juveniles. Expression of *spPHI*, uncharacterized gene *LOC105328839*, and *vasa* is relatively high in blastula but decreases significantly as larval development progresses (Fig. 2D).

### Single-cell transcriptomics highlights tissue-specific progenitors in C. gigas gastrulae

**Fig. 3:**
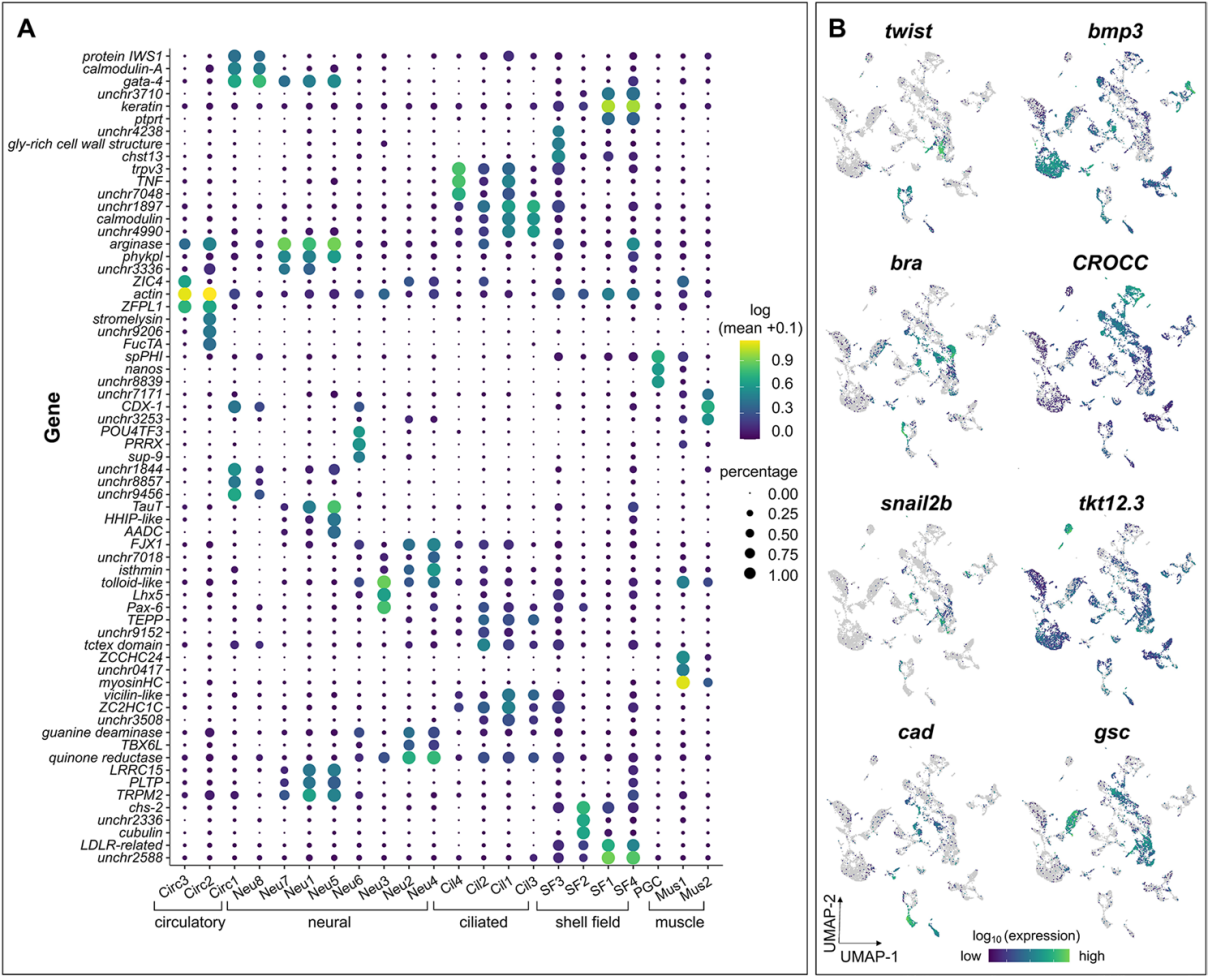
Single-cell transcriptomics highlights tissue-specific progenitors in *C. gigas* gastrulae defined by expression of evolutionarily conserved germ layer markers and novel genes identified via cluster analysis. (**A**) Dot plot illustrating expression of the top 3 marker genes (by marker_score) associated with each UMAP cluster. Dot color represents expression level and dot size notes fractional representation of cells expressing a gene. (**B**) Expression patterns of select genes associated with developmental markers in UMAP space

While gene expression atlases are not as well defined for bivalves compared to more established invertebrate developmental models like sea urchin [35, 36] and *Drosophila* [42], we were able to broadly define oyster gastrula cell clusters by assessing expression of evolutionarily conserved metazoan marker genes. These include identification of cell populations corresponding to highly ciliated cells, muscle, shell field, epidermis, neuronal precursors, and some undifferentiated cells (Fig. 3A-B; Table 1). To annotate these 27 clusters with putative cell-type information (Fig. 1C), we queried the dataset using a combination of highly conserved bilaterian marker genes and previously identified genes expressed in specific tissues or cells in other molluscs (Table 1; Fig S2; Table S6).

#### Evolutionarily conserved germ layer and ciliated cell markers are expressed in C. gigas gastrulae

Bivalve embryos and larvae are often highly ciliated, with prototroch precursors developing during the gastrula stage [61]. We identified multiple clusters representing putative ciliated cells with high levels of expression of well-established ciliary genes (Cil1-4; Table 1; Fig. 3). For example, we found that *rootletin* (CROCC (Ciliary Rootlet Coiled-Coil)) is one of the most specifically expressed genes in the ciliated cell clusters, along with *trichohyalin*, *dynein*, *tektin*, *foxJ*, and multiple tubulins, which are expressed in ciliated cell types in other bilaterian larvae [37, 38, 62, 63]. Interestingly, *caveolin*, shown to be expressed in the developing prototroch of *Dreissena* trochophore larvae [37], is one of the most specifically expressed genes in cluster Un3, which also has other ectomesodermal expression signatures (Table 1).

In *C. gigas* gastrulae, expression of mesodermal markers *bra*,* twist*, and *snail2* expression is high in portions of some undefined clusters (Un2-5), as well as some cells in shell field cluster SF2 and muscle cluster Mus1 (Fig. 3B), indicating that these clusters likely represent mesodermal derivatives. Bone morphogenetic protein 3 (*bmp3*), which is expressed in endomesodermal tissues in larval pearl oyster [64], shows widespread expression in putative endomesodermal clusters in *C. gigas* gastrulae, including portions of putative muscle precursors (Fig. 3B). Hemocyte marker transketolase-like protein 2 (*tktl2.3*) [38] is highly expressed in putative circulatory-associated cluster Circ2, with lower expression in shell field clusters (Fig. 3B). In *C. gigas* gastrulae, caudal (*cad*/*cdx*), an ectomesodermal marker in other mollusc embryos [65–67] is expressed in clusters Neu6, Circ1, Mus2, and portions of undefined clusters Un2-5 (Fig. 3B; Table 1).

#### Biomineralization and ectodermal genes define shell field progenitors

**Fig. 4:**
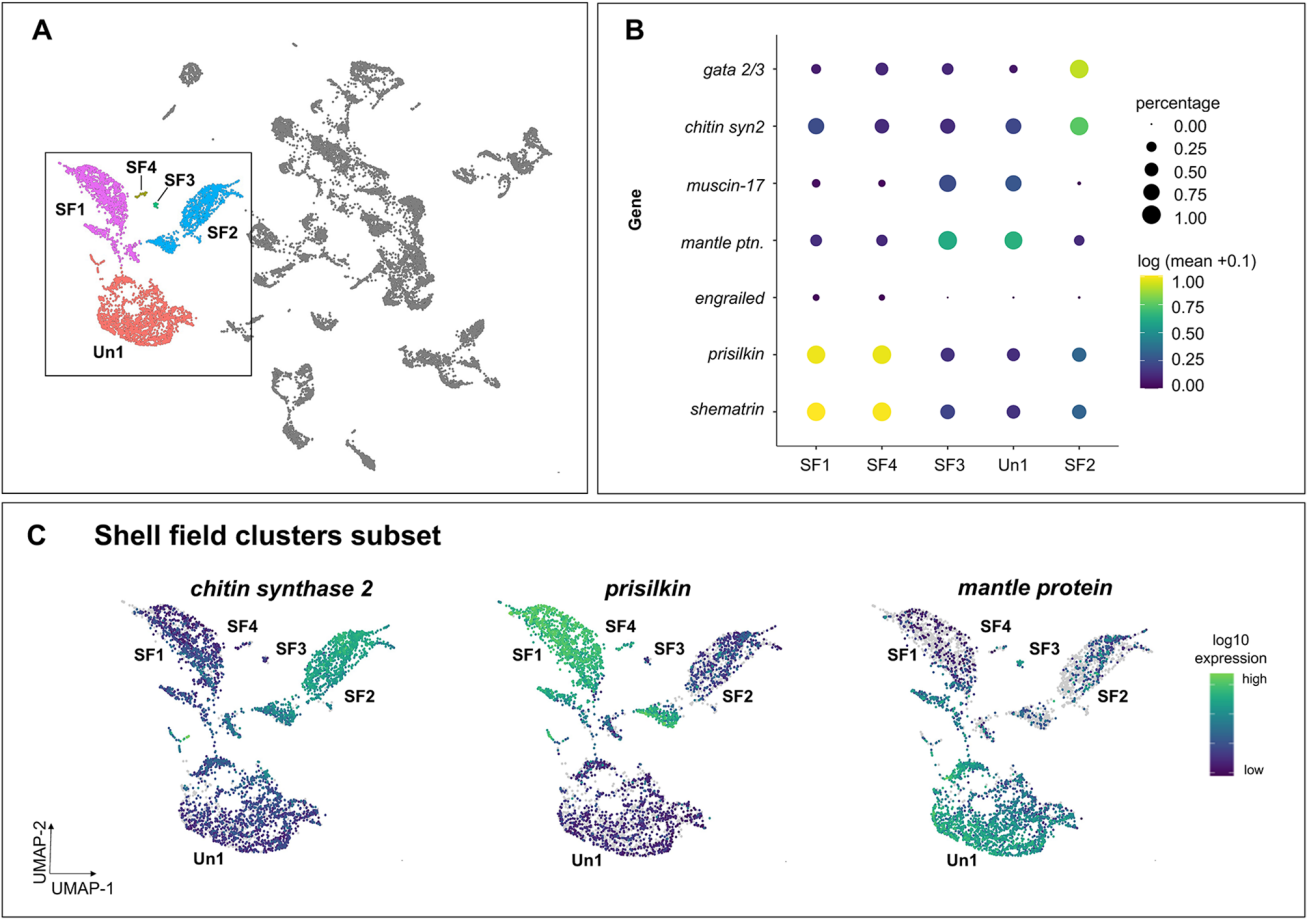
Multiple clusters expressing biomineralization and ectodermal genes suggest tissue-level shell field organization is becoming defined by the mid-gastrula stage. (**A**) UMAP color inset coded by clusters associated with the developing shell field in *C. gigas* gastrula embryos. (**B**) Dot plot illustrating expression of genes expressed in shell field subclusters. Dot color represents expression level and dot size represents fractional representation of cells expressing a gene. (**C**) Expression patterns of select genes associated with shell field clusters in UMAP space

We found that *C. gigas* gastrulae have multiple cell clusters expressing genes associated with developing shell field, with biomineralization pathway components being highly expressed in clusters SF1, SF2 and SF4 (Fig. 4; Table S2). Additionally, homeobox transcription factor goosecoid (*gsc*), which is expressed in both the adult mantle and larval shell field in gastropods [68] is highly expressed in SF2 as well as undefined clusters 2,3,5 (Fig. 3B). Gastrula shell field clusters also express *sox2*, *gata2/3*, and *engrailed*, other known genes involved in the developing shell field in *C. gigas* trochophores and veligers (Table 1) [69–71]. *Hox1*, which is expressed in the shell field of freshwater mussel trochophores [72] shows high levels of expression in clusters Un-1, as well as Neu4.

Interestingly, even at this early developmental stage, expression patterns of mantle or shell field-associated genes show differences between shell field clusters (Fig. 4B, C). For example, genes involved in chitin synthesis are highly expressed in SF2, while *shematrin* and *prisilkin-39* are expressed in SF1 and SF4 (Fig. 4B, C; Fig. S2), suggesting that these tissue-level shell field distinctions are becoming defined by the mid-gastrula stage. SF3 shows preferential expression of *mucin-17*; mucins have been shown to be involved in biomineralization processes in mussels [73]. Interestingly, *mantle protein* (LOC105326721) is expressed at a higher level in Un1 and SF3 compared to the other putative shell field clusters, though these two clusters do not have substantial expression of biomineralization genes.

#### Muscle precursors

**Fig. 5:**
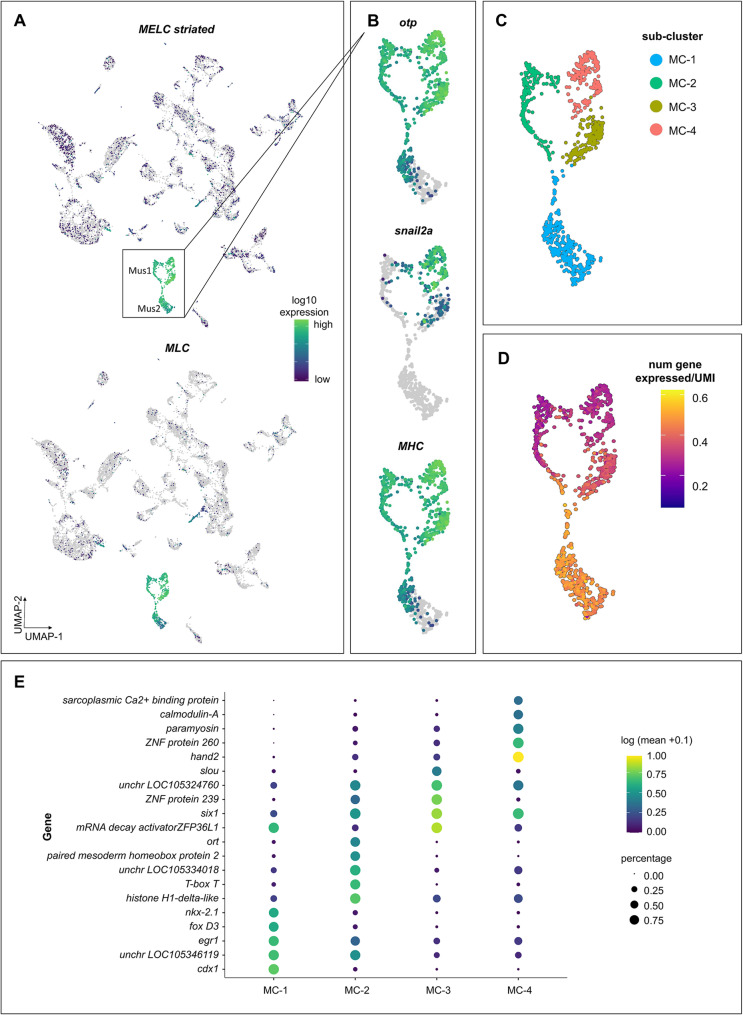
Two clusters expressing genes associated with the development of larval muscle in *C. gigas* gastrulae.** (A**) Expression of myosin essential light chain - striated muscle (*MELC striated*) and myosin light chain (*MLC*) are highest in the two putative muscle-associated clusters of gastrulae in UMAP space. (**B**) Expression of muscle-related genes *otp*, *snail2a* and *MHC* show variation in expression within each putative muscle cluster. (**C**) Sub-cluster analysis of muscle-associated clusters identified four sub-clusters. (**D**) Visualizing the number of genes expressed per cell suggests a potential developmental trajectory, with cells from cluster Mus1 representing the developmentally oldest cells. (**E**) Dot plot illustrating expression of muscle-associated marker genes of each associated sub-cluster. Dot color represents expression level and dot size represents fractional representation of cells expressing a gene

Two gastrula cell clusters show expression of known muscle marker genes including *myosin*, *troponin*, and *twist*, indicating that these clusters likely represent muscle precursors (Mus1, Mus2; Fig. 5A; Table S2). Myosin essential light chain (*MELC*; striated adductor muscle; LOC105317061), which has been demonstrated by *in-situ* hybridization to be expressed as early as blastula stage in *C. gigas* [74], is highly expressed in both clusters Mus1, Mus2 (Fig. 5A). An additional *MELC* associated with smooth adductor muscle (LOC105320997) also shows high levels of expression in the putative muscle precursor clusters. In *C. gigas* gastrulae, *twist*, a basic helix-loop-helix (bHLH) transcription factor essential for development of the mesoderm, is expressed in cluster Mus1, with highest expression observed in portions of putative ectomesodermal cluster Un3 (Fig. 3B). Within the clusters of muscle precursors, there appear to be some further separation of cells based on gene expression differences. For example, *otp* is expressed in only half of cluster Mus1 (Fig. 5B). Myosin heavy chain (*mhc*, LOC105338907) is highly expressed in cluster Mus1, with some expression of adjacent cells in cluster Mus2 (Fig. 5B).

To assess developing muscle cell progression through the differentiation process, we performed focused clustering and pseudotime analyses of clusters Mus1 and EnM-Mus2. These two clusters subsequently yielded four sub-clusters (MC 1–4) and a potential developmental trajectory based on the number of genes expressed per cell, which can be used as a proxy for developmental progress [75] (Fig. 5C-D). Muscle cluster-1 is defined by high expression of transcription factors associated with endomesodermal and muscle specification (Fig. 5E), consistent with an analysis showing a relatively higher number of genes expressed per cell suggestive of a “younger” developmental age [75] (Fig. 5D). Mus3 shows slightly fewer genes expressed per cell compared to Mus1, but cells in this cluster also express genes associated with muscle tissue differentiation. For example, a homolog of the *Drosophila* gene *slouch* (LOC105345169) - a homeodomain-containing protein involved in muscle specification [76] - is one of the defining marker genes for Mus3 (Fig. 5E).

#### Putative neural and sensory cell precursors

**Fig. 6:**
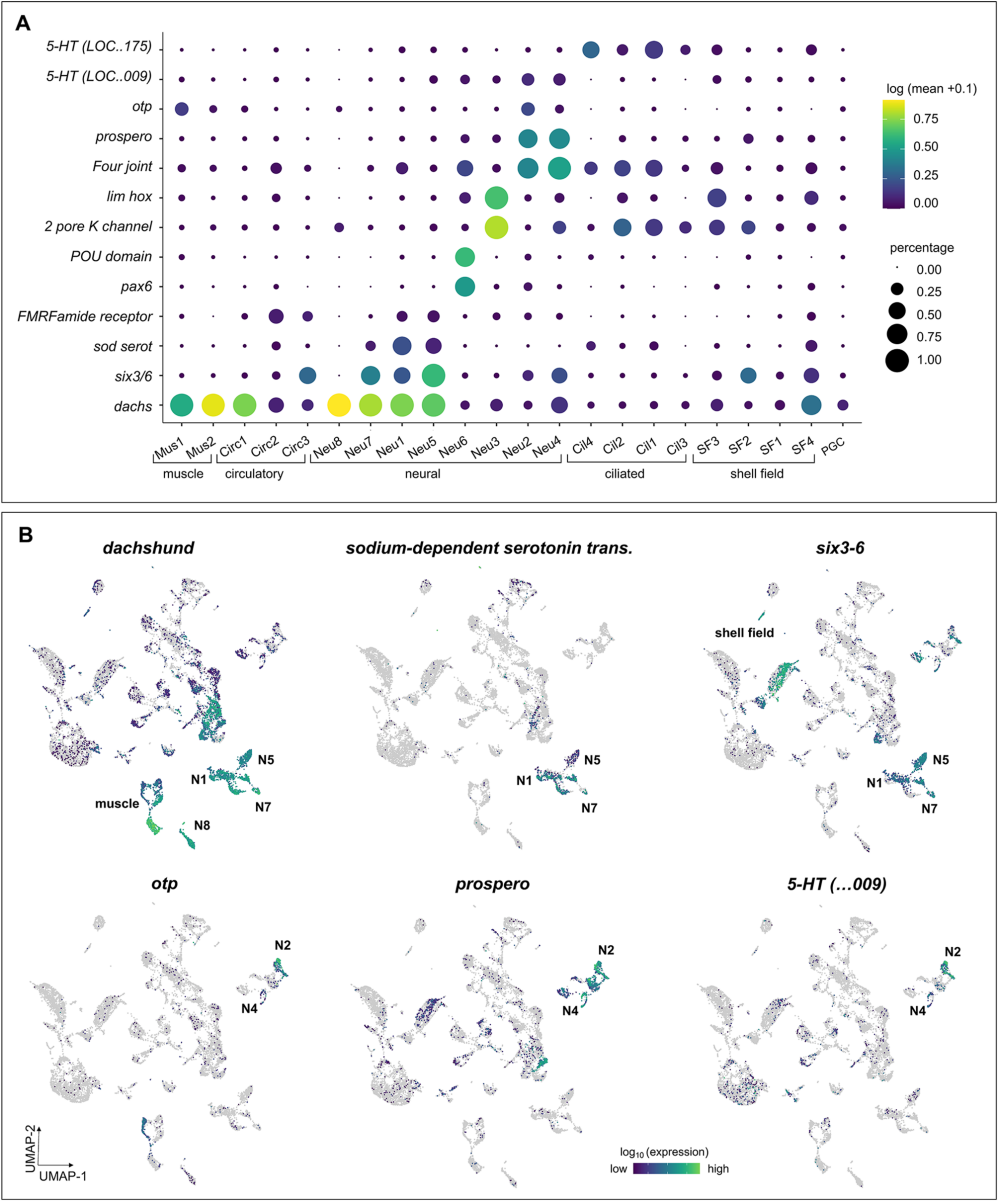
Multiple cell clusters expressing conserved neuronal or sensory-related genes are present in *C. gigas* gastrulae. Dot plot (**A**) and UMAP (**B**) representation of expression of select genes associated with neural clusters. For the dot plot and UMAPs, dot color represents expression level, and for the dot plot, dot size represents fractional representation of cells expressing a gene. For the UMAP, clusters with a high proportion of cells expressing a given gene are labeled for orientation

In gastrula-stage samples, we identified multiple cell clusters expressing conserved neuronal or sensory-related genes (Neu1-8; Fig. 6; Table 1). Strikingly, we detected disparate clusters of putative neural or sensory cells based on distinct patterns of neural gene expression, even at this relatively early embryonic stage (Fig. 6). These include clusters of cells expressing the neuronal transcription factor *prospero* (highest in clusters Un3, Neu2, Neu4) [77], four-jointed box kinase (*four-jointed*; clusters Neu2, Neu4, Cil1, Cil2) [78], *sodium-dependent serotonin transporter* (clusters Neu1, Neu5, Neu7), and the neural and eye-associated nuclear protein *dachshund* (clusters Neu1, Neu5, Neu7, Neu8, as well as muscle and some portions of undefined clusters) [79].

The 5-hydroxytryptamine receptor (*5-HTR*; LOC105348009, LOC105345175), associated with the developing apical organ in oyster trochophores [80], shows high levels of expression in clusters Neu1, Neu4, and Cil1 (Fig. 6A-B). Orthopedia (*otp*) is a homeodomain-containing transcription factor expressed during nervous system development in diverse clades including molluscs [65, 81]. Interestingly, we detected *otp* expression in only one neural-associated cluster (Neu2). We found that homeodomain-containing transcription factor *six3/6* is expressed in most predicted neuronal or sensory cell clusters, as well as in some shell field clusters (Fig. 6).

The *FMRFamide receptor* gene shows some expression in our gastrulae dataset, though at relatively low levels (Fig. 6A; Fig. S4). Some known neural genes such as *hex* (LOC105339244) are expressed in other mollusc larvae but were not detected in our cleavage + blastula (Fig. S4) or gastrula datasets, possibly because these genes are not expressed until later in larval development.

### Early signatures of transcriptional states of cleavage-stage and blastula embryos

**Fig. 7:**
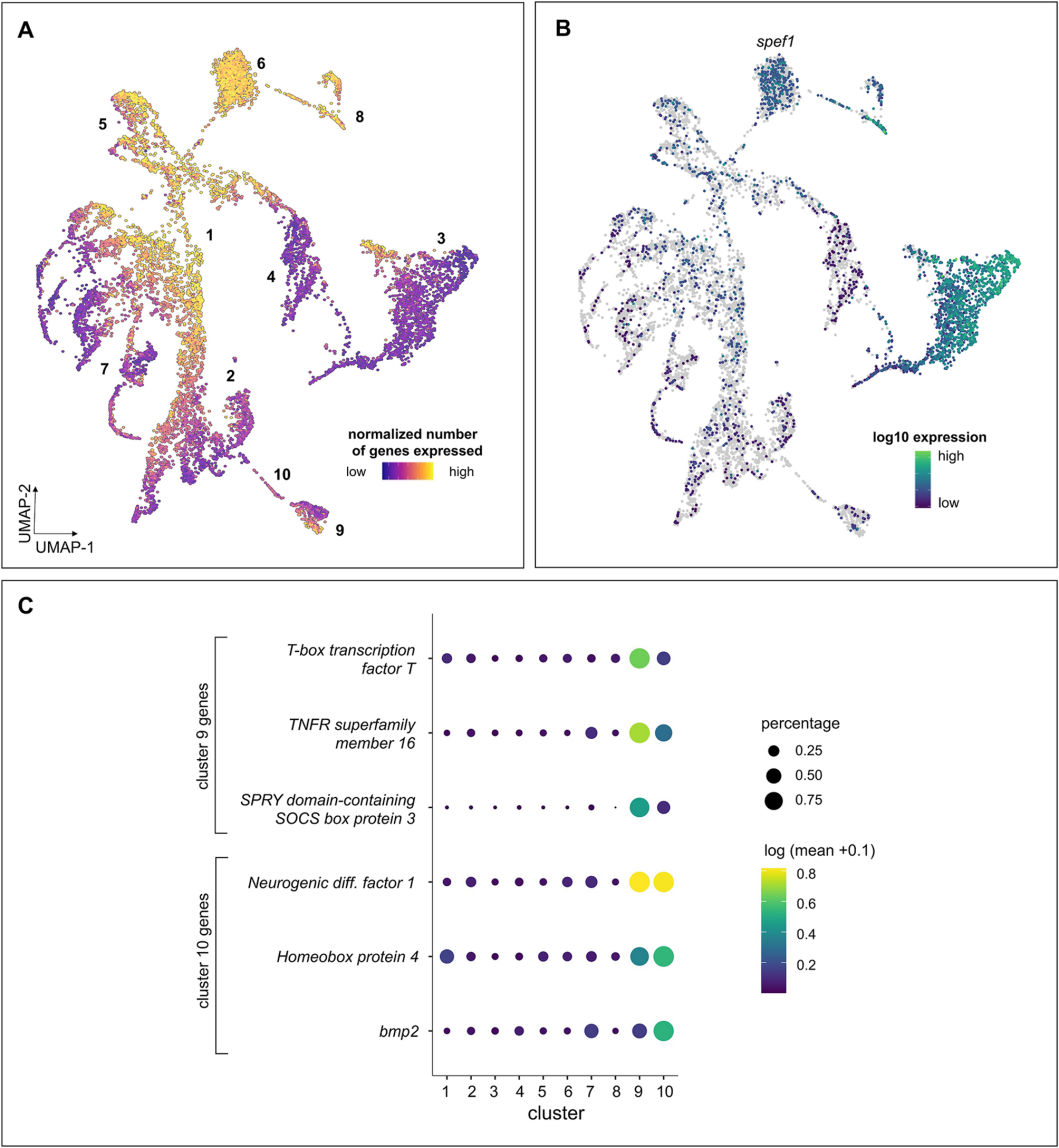
Early signatures of transcriptional states of cleavage-stage and blastula embryos, representing a continuum of the earliest stages of embryonic development in *C. gigas.***(A**) UMAP of cleavage + blastula color-coded by the normalized number of genes expressed per cell (i.e. number of genes expressed/number of UMI per cell). (**B**) UMAP representation of expression of select gene associated with the ciliated cluster of cleavage + blastula cells. (**C**) Dot plot representation of expression of top 3 marker genes associated with cluster 9 and 10 of the cleavage + blastula cells. Dot color represents expression level and for the dot plot dot size represents fractional representation of cells expressing a gene

Developmental ordering of cells from cleavage-stage and blastula embryos, which represent a continuum of the earliest stages of embryonic development, showed that we could identify a developmental progression of cells that is consistent with their transcriptional continuity in low dimensional space. Visualization of cells expressing the fewest genes (which are predicted to be the most differentiated cells relative to progenitors [75]) (Fig. 7A) and by visualization of the blastula cells in UMAP space (Fig. S5), showed that cluster cb_9 and portions of cluster cb_3 are likely to be some of the developmentally oldest cells. While cell-type cluster annotation is challenging at this early developmental stage, top marker analysis showed signatures of ciliated cells in cluster cb_3, which were expressing genes such as sperm flagellar protein 1 (*spef1*; LOC105339310) and EF-hand calcium binding domain-containing protein 6 (*efcab6*; LOC105337196) (Fig. 7B, Table S3). Additionally, a marker gene analysis showed clusters cb_9 and cb_10 had the highest specificity of all the clusters (Table S3) and many of these specific genes were transcription factors or other regulatory proteins (Fig. 7C, Table S3). Blastula cells were also analyzed separately. Clustering analysis identified 8 distinct clusters (Fig. S6). Marker gene analysis of blastulae only shows enrichment for diverse transcription factor families (Table S4), an indication of embryonic genome activation [30, 82, 83].

## Discussion

Sterile shellfish are both a market-driven need and an ecologically sustainable approach to increase food production via aquaculture. Technological advances in sterility induction, via inactivation of genes essential for germ cell formation and development in finfish aquaculture [1, 3, 23, 24], have poised the shellfish industry to incorporate this approach, but the lack of knowledge of the genes essential for PGC specification remains a significant barrier. To overcome this limitation, we have applied scRNA-seq to early stages of Pacific oyster embryos to identify genes involved in PGC specification. We present a robust suite of early developmental gene expression data using scRNA-seq in *C. gigas*. In gastrulae, though no discrete tissues are formed and the trochophore body plan is not yet defined, we identified cell clusters expressing evolutionarily conserved marker genes associated with bivalve larval tissues or cell types including muscle, shell field, neuronal cells, ciliated cells, and PGCs. We show that the molecular identity of oyster PGCs is defined by gastrula-stage, and identified and localized a number of novel genes expressed in these putative PGCs. These data also provide a rich transcriptional atlas that can be mined to address both basic and applied research questions in Pacific oyster, an emerging molluscan model species with significant commercial and ecological importance.

### Characterization of genes associated with PGC formation

We were particularly interested in the unique transcriptomic signatures associated with formation of PGCs in *C. gigas*. The early developmental stages sequenced in this study were selected to focus on initial characterization of genes potentially involved in PGC specification, a biological process that occurs very early in embryonic development. Using the conserved germline marker genes *vasa* and *nanos* to initially select cells in oyster gastrula that likely represent PGCs, we then detected transcriptomic signatures in these cells that include both novel and previously described germline-associated genes in other invertebrates.

A gene highly expressed in the PGC cluster is a histone variant, sperm-specific protein PHI-2B/PHI-3 (*spPHI*), which has not been functionally characterized in *C. gigas* but exhibits sequence similarity to a protamine-like gene uniquely expressed in spermatozoa of *Mytilus californianus* [60]. It is interesting to consider the role for a protamine-like or H1 linker gene in germ cell specification in *C. gigas* as many animals have embryo and germline-specific H1 linker proteins [84]. In *C. gigas*, *spPHI* is broadly expressed in the earliest embryos, but its highest expression is restricted to the PGC cluster by gastrulation. This pattern is similar to expression patterns in *Drosophila*, in which the embryonic H1 (*dBigH1*) gene is expressed only in the germline, where it acts to maintain transcriptional quiescence [84]. A thorough spatial, temporal, and functional characterization of the *spPHI* transcripts in *C. gigas* is necessary to determine the role of *spPHI* in embryogenesis and germline formation in oysters. The gene fem-1 homolog A-A (*fem-1*), a stem-loop binding protein involved in processing translation and degradation of replication dependent histone mRNAs, is also expressed in the PGC cluster (Fig. 2A; Table 2). First identified in *Caenorhabditis elegans*, *fem-1* plays a vital role in sex determination in diverse taxa, including bivalves [85–87]. Also specifically expressed in the oyster gastrula PGC cluster is structural maintenance of chromatin protein 6 (*smc6*) (Fig. 2A; Table 2), which has been shown to play a role in maintaining germline integrity in *C. elegans* [88]. Similarly, ATRX chromatin remodeler (*ATRX*) is expressed highly in female gonad tissue in another oyster species, *Crassostrea hongkongensis* [89].

In addition to known candidate genes involved in germline identity in other animals, this study sought to identify mollusc- or oyster-specific PGC genes that may not have been detected using a candidate approach. Indeed, an uncharacterized gene *LOC105328839* has the highest expression specificity to the PGC cluster in *C. gigas* gastrulae. This gene appears to be bivalve-specific and has few conserved protein domains that may suggest a specific molecular characterization and role in the embryo. Importantly, for both s*pPHI* and *LOC105328839*, we show via semi-quantitative PCR that expression peaks early in development, prior to the larval trochophore stage and remains relatively low through the juvenile stage, suggesting their embryonic functions may be exclusively associated with the developing germline.

Interestingly, with the exception of *nanos* and *vasa*, our study did not identify many previously identified gonad-specific genes reported in a single-cell sequencing analysis of *C. gigas* adult gonadal tissue [44]. It is possible that there may be few shared genes involved in both embryonic PGC specification and adult germinal stem cell populations in *C. gigas*, although differences in methodologies and detection between studies (i.e. sequencing depth) may be a confounding factor [90]. Nevertheless, our identification of genes associated with PGCs in *C. gigas* may help with future characterization of GSCs and processes related to gametogenesis in oysters.

In addition to providing information about potential genes involved in PGC specification in bivalves, the experimental design employed here allows for some insight into mechanisms of specification. There is a general lack of consensus in the literature on whether molluscan PGCs are specified via preformation or epigenesis [91, 92]. At the molecular level, previous work in *C. gigas* showed *vasa* expression to be asymmetrically localized in cleavage-stage embryos, suggesting that the oyster germline is specified via preformation [25]. Our results are generally consistent with that supposition, as *vasa* expression is widespread in cleavage and blastula-stage embryos; however, cells with the highest abundance of *vasa* transcripts are concentrated within a smaller region of cluster 2 (Fig. 2). These *vasa* transcripts potentially represent maternal transcripts undergoing sequestration to the presumptive germline. More recently, however, a study evaluating embryonic expression of *nanos* in Pacific oysters led to speculation that the process of formation of PGCs in *C. gigas* may involve elements of both epigenesis and preformation, perhaps in an evolutionarily transitional state [59].

In our study, *nanos* expression was highly specific to the PGC cluster in gastrulae but *not* highly expressed in any cluster of the cleavage-blastula stages. Other evidence, such as the expression of PGC cluster-specific genes, suggests PGC formation in oysters is occurring via induction of signaling and transcriptomic activity from the embryo. Interestingly, even in well-characterized model systems such as *Drosophila*, the traditional paradigm of exclusivity with regards to whether preformation or epigenesis yields formation of PGCs is being questioned as new data emerge suggesting both maternal and inductive signals are important [30]. While the results here represent a transcriptional snapshot in developmental time, and we cannot definitively resolve the mechanism of PGC formation in *C. gigas*, they imply the importance of both maternal and zygotically-derived cell differentiation factors.

### Early signatures of larval tissue formation in C. gigas

Gastrulation is a fundamental morphogenic process during early development that results in a reorganization of a hollow epithelial ball of cells into a multilayered embryo, and establishes the generation of tissues and cell types preceding organogenesis. Gastrulae sequencing detected expression of conserved genes involved in germ layer specification, a morphogenetic and intensive cell signaling event that occurs during gastrulation to define germ layers - endoderm, ectoderm, mesoderm - and leads to the establishment of the gut [93, 94]. In spiralian lophotrochozoan species like oysters, mesoderm develops from both ectodermal and endodermal-associated bipotential precursor cells, with ectomesodermal cells located near the blastopore [65]. We found gene expression signatures in gastrulae coinciding with the establishment of germ layers and tissue types. For example, brachyury (*bra*) is a conserved T-box transcription factor with mesodermal expression across bilaterian taxa, including in the gastropod *Crepidula* [65] and the mussel *Dreissena* [95], as well as in the developing shell field of gastropod larvae [96, 97]. We detected *bra* most highly in “undefined” clusters and portions of shell field and muscle clusters, indicating that these represent developing endomesoderm or mesodermal derivatives. Similarly, caudal (*cad/cdx*), a homeobox-containing transcription factor important for gastrulation, anterior/posterior axis specification, and shown previously to be expressed in ectodermal and ectomesodermal cells in mollusc embryos [65–67], was expressed primarily in undefined and portions of one of the muscle clusters. Some typical mesodermal genes like *hes* and *mox* [98] were not detected in any of our datasets, possibly due to the early developmental stages targeted. In bivalve molluscs, specific cell types are defined by an early larval stage - the trochophore - but the gene expression signatures preceding the establishment of this larval body plan during gastrulation, including those associated with neural, muscle, shell-secretion, and primordial germ cells are not well established. In invertebrate gastrulae, single-cell sequencing has been an effective technique to identify cell-type differentiation trajectories, discover novel genes associated with developing tissues, and better understand gene regulatory networks [35, 36, 42, 43]. We identified putative cell clusters associated with multiple developing larval tissues or cell types, including ciliated cells, muscles, sensory or neural cells, and shell gland.

Gastrulae sequencing data revealed several distinct cell clusters that express genes known to be involved in mollusc shell formation [99]. Mollusc shells are composed primarily of calcium carbonate (CaCO3), with smaller proportions of organic compounds like chitin and glycoproteins (reviewed in [100]). Larval molluscs have a structure called the shell field or shell gland that secretes shell material [101, 102]. The shell field first appears as a visually identifiable structure during the gastrula stage as a thickened cap of ectoderm on the embryo’s dorsal side [103]. Genes involved in synthesis of mollusc shell matrix have been identified in *C*. *gigas* [104] and shown to be expressed in late trochophore larvae [38].

Even at this early developmental stage, we identified four clusters associated with the developing shell field as well as a fifth cluster that highly expresses *mantle protein* (Fig. 4). Our findings are consistent with the results of Liu et al. [105], who identified multiple defined cell populations from the shell gland of *C. gigas* gastrulae using in-situ hybridization for individual known mollusc mantle genes. Embryonic insights into the sequestration of distinct regions of developing mantle tissues can inform evolutionary mechanisms driving the observed array of mollusc shell morphologies [99, 106, 107]. Additionally, these data can inform how larval shell formation may be perturbed by environmental stressors, such as ocean acidification. Ocean acidification can impact many physiological systems in marine invertebrates, but larval shell formation has been shown to be particularly sensitive [108]. Transcriptomic studies of whole bivalve larvae have hinted at mechanisms underlying developmental delays observed upon ocean acidification exposure [109, 110]. The data provided here, at single-cell resolution, could provide additional information to identify potential transcriptomic targets to gain further insights into this process.

The oyster larval nervous system – an apical sensory organ and some identifiable nerves - begins to appear during the trochophore stage, though trochophore larvae possess fewer defined neural structures compared to the later veliger larval stage [38, 111, 112]. Here we identified multiple clusters in *C. gigas* gastrulae that are expressing genes known to be present in early peripheral sensory cells and other neural progenitors, including *5-hydroxytryptamine receptor* and *otp*, which are expressed in cells associated with the apical organ in oyster trochophores [80, 81]. The homeodomain-containing transcription factor *six3/6*, which we found in this study is expressed in some gastrulae neural-associated clusters, is involved in the development of sensory structures in diverse metazoans, and has been shown to be expressed in the neural structures of mollusc embryos and larvae [65, 113]. Planktonic larvae, particularly in species with feeding (planktotrophic) larvae such as Pacific oyster, must be able to sense their environment, control swimming direction through phototaxis and gravity sensing, and locate prey. It is perhaps not unexpected that even at the gastrula stage, gene expression for developing sensory cells and neurons was detected in our data.

### Oyster gastrulae may inform evolutionary questions about developmental processes during embryogenesis

Collectively, our data represent transcriptomes from the earliest developmental time-points sequenced at the single-cell level in a lophotrochozoan species. This single-cell resolution during major early developmental milestones can potentially reveal the transcriptional signatures within trajectories of cell state transitions that lead to the lineage commitment of germ layers and discrete tissue types in an evolutionarily fascinating model bivalve mollusc. Additionally, gene expression insights from oyster gastrula may be useful for studies exploring the comparative evolution of developmental processes and tissue patterning both within lophotrochozoans and between ecdysozoan taxa and deuterostomes. By identifying genes uniquely expressed within each grouping of putative differentiating cell or tissue types including both evolutionarily conserved marker genes and uncharacterized mollusc-specific genes, these data can be a resource for pairing newly discovered genes with those known to be involved in cell-type or tissue information. Indeed, similar efforts have yielded insights into the genes driving embryonic and larval body plan patterning in other invertebrates, including detection of previously elusive progenitor cells [38, 63, 114].

## Conclusions

These sequencing data and cell-type atlases provide a rich dataset exploring transcriptional activities and cell-type differentiation during early oyster development. The gastrula single-cell atlas presented here should also inform future studies exploring cell-type identity and developmental trajectories in larval bivalves. Notably, we have identified a suite of candidate genes that can be explored for their role in oyster PGC specification and to advance efforts to develop methods to achieve reproductive sterility via germ cell elimination in cultured Pacific oysters and potentially other shellfish species. Taken together, this dataset shows the utility of scRNA-seq to describe cell type differentiation and PGC specification during mollusc development, a group that has been understudied from this perspective.

## Supplementary Information

Supplementary Tables

Supplementary Figures

## Data Availability

Sequence data that support the findings of this study have been deposited into the NCBI SRA database with the primary accession code PRJNA906172.

## References

[1] Wargelius A, Leininger S, Skaftnesmo KO, Kleppe L, Andersson E, Taranger GL, Schulz RW, Edvardsen RB. Dnd knockout ablates germ cells and demonstrates germ cell independent sex differentiation in Atlantic salmon. Sci Rep. 2016;6(1):21284. 26888627 10.1038/srep21284 PMC4758030

[2] Kleppe L, Fjelldal PG, Andersson E, Hansen T, Sanden M, Bruvik A, Skaftnesmo KO, Furmanek T, Kjærner-Semb E, Crespo D, Flavell S. Full production cycle performance of gene-edited, sterile Atlantic salmon-growth, smoltification, welfare indicators and fillet composition. Aquaculture. 2022;560:738456.

[3] Xu L, Zhao M, Zohar Y, Wong TT. Induction of reproductive sterility in coho salmon (*Oncorhynchus kisutch*) by an immersion-based gene silencing technology. J Mar Sci Eng. 2023;11(12):2208.

[4] Xu L, Zhao M, Ryu JH, Hayman ES, Fairgrieve WT, Zohar Y, et al. Reproductive sterility in aquaculture: a review of induction methods and an emerging approach with application to Pacific Northwest finfish species. Rev Aquacult. 2023;15(1):220–41.

[5] Nell JA. Farming triploid oysters. Aquaculture. 2002;210(1–4):69–88.

[6] Piferrer F, Beaumont A, Falguière JC, Flajšhans M, Haffray P, Colombo L. Polyploid fish and shellfish: production, biology and applications to aquaculture for performance improvement and genetic containment. Aquaculture. 2009;293(3–4):125–56.

[7] Thunberg. 1793.

[8] Salvi D, Macali A, Mariottini P. Molecular phylogenetics and systematics of the bivalve family *Ostreidae* based on rRNA sequence-structure models and multilocus species tree. PLoS ONE. 2014;9(9):e108696. 25250663 10.1371/journal.pone.0108696 PMC4177229

[9] Bayne BL, Ahrens M, Allen SK, D’auriac MA, Backeljau T, Beninger P, et al. The proposed dropping of the genus *Crassostrea* for all Pacific cupped oysters and its replacement by a new genus *Magallana*: a dissenting view. J Shellfish Res. 2017;36(3):545–7.

[10] Agriculture Organization of the United Nations. Fisheries Department. The state of world fisheries and aquaculture. Food and Agriculture Organization of the United Nations; 2018.

[11] Robledo JA, Yadavalli R, Allam B, Espinosa EP, Gerdol M, Greco S, et al. From the raw bar to the bench: bivalves as models for human health. Dev Comp Immunol. 2019;92:260–82. 30503358 10.1016/j.dci.2018.11.020 PMC6511260

[12] Grabowski JH, Peterson CH. Restoring oyster reefs to recover ecosystem services. Theoretical Ecol Ser. 2007;4(101016):80017–7.

[13] Downing SL, Allen SK Jr. Induced triploidy in the Pacific oyster, *Crassostrea gigas*: optimal treatments with cytochalasin B depend on temperature. Aquaculture. 1987;61(1):1–5.

[14] Guo X, Wang Y, Xu Z, Yang H. Chromosome set manipulation in shellfish. New technologies in aquaculture. Woodhead Publishing. 2009;165–94.

[15] Allen SK, Downing SL. Performance of triploid Pacific oysters, *Crassostrea gigas* (Thunberg). i. Survival, growth, glycogen content, and sexual maturation in yearlings. J Exp Mar Biol Ecol. 1986;102(2–3):197–208.

[16] Allen SJ, Downing SL. Consumers and experts alike prefer the taste of sterile triploid over gravid diploid Pacific oysters (*Crassostrea gigas*, Thunberg, 1793). J Shellfish Res. 1991;10(1):19–22.

[17] Degremont L, Garcia C, Frank-Lawale A, Allen SK. Triploid oysters in the Chesapeake Bay: comparison of diploid and triploid *Crassostrea virginica*. J Shellfish Res. 2012;31(1):21–31.

[18] Normand J, Le Pennec M, Boudry P. Comparative histological study of gametogenesis in diploid and triploid Pacific oysters (*Crassostrea gigas*) reared in an estuarine farming site in France during the 2003 heatwave. Aquaculture. 2008;282(1–4):124–9.

[19] Shpigel M, Barber BJ, Mann R. Effects of elevated temperature on growth, gametogenesis, physiology, and biochemical composition in diploid and triploid Pacific oysters, *Crassostrea gigas* Thunberg. J Exp Mar Biol Ecol. 1992;161(1):15–25.

[20] Houssin M, Trancart S, Denechere L, Oden E, Adeline B, Lepoitevin M, Pitel PH. Abnormal mortality of triploid adult Pacific oysters: is there a correlation with high gametogenesis in Normandy, france? Aquaculture. 2019;505:63–71.

[21] Wadsworth P, Casas S, La Peyre J, Walton W. Elevated mortalities of triploid Eastern oysters cultured off-bottom in Northern Gulf of Mexico. Aquaculture. 2019;505:363–73.

[22] Guévélou E, Carnegie RB, Small JM, Hudson K, Reece KS, Rybovich MM. Tracking triploid mortalities of Eastern oysters *Crassostrea Virginica* in the Virginia portion of the Chesapeake Bay. J Shellfish Res. 2019;38(1):101–13.

[23] Wong TT, Zohar Y. Production of reproductively sterile fish by a non-transgenic gene silencing technology. Sci Rep. 2015;5(1):15822. 26510515 10.1038/srep15822 PMC4625178

[24] Yanagitsuru YR, Hayman ES, Fairgrieve WT, Zohar Y, Wong TT, Luckenbach JA. Proof-of-concept for sterility induction in sablefish (Anoplopoma fimbria) via a scalable immersion-based gene silencing approach. Aquaculture. 2025;9:742945.

[25] Fabioux C, Pouvreau S, Le Roux F, Huvet A. The oyster vasa-like gene: a specific marker of the germline in *Crassostrea gigas*. Biochem Biophys Res Commun. 2004;315(4):897–904. 14985097 10.1016/j.bbrc.2004.01.145

[26] Fabioux C, Huvet A, Lelong C, Robert R, Pouvreau S, Daniel JY, et al. Oyster vasa-like gene as a marker of the germline cell development in *Crassostrea gigas*. Biochem Biophys Res Commun. 2004;320(2):592–8. 15219870 10.1016/j.bbrc.2004.06.009

[27] Fabioux C, Corporeau C, Quillien V, Favrel P, Huvet A. In vivo RNA interference in oyster–vasa silencing inhibits germ cell development. FEBS J. 2009;276(9):2566–73. 19476495 10.1111/j.1742-4658.2009.06982.x

[28] Xu L, Small JM, Hood SM, Zhao M, Plough LV, Wong TT. Morpholino oligomer delivery via bath immersion for use in reverse genetic studies on the early development of Eastern oysters (*Crassostrea virginica*). Aquaculture. 2025;5:742261.

[29] Gustafson EA, Wessel GM. Vasa genes: emerging roles in the germ line and in multipotent cells. Bioessays. 2010;32(7):626-37. 10.1002/bies.201000001 PMC3090673 20586054

[30] Colonnetta MM, Schedl P, Deshpande G. Germline/soma distinction in Drosophila embryos requires regulators of zygotic genome activation. Elife. 2023;12:e78188. 10.7554/eLife.78188 PMC9812407 36598809

[31] Miramón-Puértolas P, Pascual-Carreras E, Steinmetz PR. A population of Vasa2 and Piwi1 expressing cells generates germ cells and neurons in a sea anemone. Nat Commun. 2024;15(1):8765. 39384751 10.1038/s41467-024-52806-4 PMC11464780

[32] Weidinger G, Stebler J, Slanchev K, Dumstrei K, Wise C, Lovell-Badge R, Thisse C, Thisse B, Raz E. Dead end, a novel vertebrate germ plasm component, is required for zebrafish primordial germ cell migration and survival. Curr Biol. 2003;13(16):1429–34. 12932328 10.1016/s0960-9822(03)00537-2

[33] Extavour CG. Evolution of the bilaterian germ line: lineage origin and modulation of specification mechanisms. Integr Comp Biol. 2007;47(5):770–85. 21669758 10.1093/icb/icm027

[34] Nakamura A, Shirae-Kurabayashi M, Hanyu-Nakamura K. Repression of early zygotic transcription in the germline. Curr Opin Cell Biol. 2010;22(6):709–14. 20817425 10.1016/j.ceb.2010.08.012

[35] Foster S, Oulhen N, Wessel G. A single cell RNA sequencing resource for early sea urchin development. Development. 2020;147(17):dev191528. 32816969 10.1242/dev.191528 PMC7502599

[36] Massri AJ, Greenstreet L, Afanassiev A, Berrio A, Wray GA, Schiebinger G, et al. Developmental single-cell transcriptomics in the *Lytechinus variegatus* sea urchin embryo. Development. 2021;148(19):dev198614. 34463740 10.1242/dev.198614 PMC8502253

[37] Salamanca-Díaz DA, Schulreich SM, Cole AG, Wanninger A. Single-cell RNA sequencing atlas from a bivalve larva enhances classical cell lineage studies. Front Ecol Evol. 2022;9:783984.

[38] Piovani L, Leite DJ, Yañez Guerra LA, Simpson F, Musser JM, Salvador-Martínez I, et al. Single-cell atlases of two lophotrochozoan larvae highlight their complex evolutionary histories. Sci Adv. 2023;9(31):eadg6034. 37531419 10.1126/sciadv.adg6034 PMC10396302

[39] Cao J, Packer JS, Ramani V, Cusanovich DA, Huynh C, Daza R, Qiu X, Lee C, Furlan SN, Steemers FJ, Adey A. Comprehensive single-cell transcriptional profiling of a multicellular organism. Science. 2017;357(6352):661–7. 28818938 10.1126/science.aam8940 PMC5894354

[40] Packer JS, Zhu Q, Huynh C, Sivaramakrishnan P, Preston E, Dueck H, et al. A lineage-resolved molecular atlas of *C. elegans* embryogenesis at single-cell resolution. Science. 2019;365(6459):eaax1971. 31488706 10.1126/science.aax1971 PMC7428862

[41] Sladitschek HL, Fiuza UM, Pavlinic D, Benes V, Hufnagel L, Neveu PA. Morphoseq: full single-cell transcriptome dynamics up to gastrulation in a chordate. Cell. 2020;181(4):922–35. 32315617 10.1016/j.cell.2020.03.055 PMC7237864

[42] Sakaguchi S, Mizuno S, Okochi Y, Tanegashima C, Nishimura O, Uemura T, et al. Single-cell transcriptome atlas of *Drosophila* gastrula 2.0. Cell Rep. 2023. 10.1016/j.celrep.2023.112707. 37433294 10.1016/j.celrep.2023.112707

[43] Satoh N, Hisata K, Foster S, Morita S, Nishitsuji K, Oulhen N, Tominaga H, Wessel GM. A single-cell RNA-seq analysis of Brachyury-expressing cell clusters suggests a morphogenesis-associated signal center of oral ectoderm in sea urchin embryos. Dev Biol. 2022;483:128–42. 35038441 10.1016/j.ydbio.2022.01.005

[44] Wang H, Yu H, Li Q. Integrative analysis of single-nucleus RNA-seq and bulk RNA-seq reveals germline cells development dynamics and niches in the Pacific oyster gonad. iScience. 2024. 10.1016/j.isci.2024.109499. 38571762 10.1016/j.isci.2024.109499 PMC10987912

[45] de La Forest Divonne S, Pouzadoux J, Romatif O, Montagnani C, Mitta G, Destoumieux-Garzon D, Gourbal B, Charrière GM, Vignal E. Diversity and functional specialization of oyster immune cells uncovered by integrative single cell level investigations. BioRxiv. 2024;23:2024–07. 10.7554/eLife.102622 PMC12064177 40343849

[46] Strathmann MF. Reproduction and development of marine invertebrates of the Northern Pacific coast: data and methods for the study of eggs, embryos, and larvae. University of Washington; 1987.

[47] Zheng GX, Terry JM, Belgrader P, Ryvkin P, Bent ZW, Wilson R, et al. Massively parallel digital transcriptional profiling of single cells. Nat Commun. 2017;8(1):14049. 28091601 10.1038/ncomms14049 PMC5241818

[48] Cao J, Spielmann M, Qiu X, Huang X, Ibrahim DM, Hill AJ, Zhang F, Mundlos S, Christiansen L, Steemers FJ, Trapnell C. The single-cell transcriptional landscape of mammalian organogenesis. Nature. 2019;566(7745):496–502. 30787437 10.1038/s41586-019-0969-x PMC6434952

[49] McInnes L, Healy J, Melville J. UMAP: Uniform Manifold Approximation and Projection for dimension reduction. 2018. Preprint at https://arxiv.org/abs/1802.03426

[50] Haghverdi L, Lun A, Morgan M, Marioni JC. Batch effects in single-cell RNA-sequencing data are corrected by matching mutual nearest neighbors. Nat Biotechnolology. 2018;36:421–7. 10.1038/nbt.4091 PMC6152897 29608177

[51] Trapnell C, Cacchiarelli D, Grimsby J, Pokharel P, Li S, Morse M, Lennon NJ, Livak KJ, Mikkelsen TS, Rinn JL. The dynamics and regulators of cell fate decisions are revealed by pseudotemporal ordering of single cells. Nat Biotechnol. 2014;32(4):381–6. 24658644 10.1038/nbt.2859 PMC4122333

[52] Altschul SF, Gish W, Miller W, Myers EW, Lipman DJ. Basic local alignment search tool. J Mol Biol. 1990;215(3):403–10. 2231712 10.1016/S0022-2836(05)80360-2

[53] Schindelin J, Arganda-Carreras I, Frise E, Kaynig V, Longair M, Pietzsch T, Preibisch S, Rueden C, Saalfeld S, Schmid B, Tinevez JY. Fiji: an open-source platform for biological-image analysis. Nat Methods. 2012;9(7):676–82. 22743772 10.1038/nmeth.2019 PMC3855844

[54] Choi HM, Schwarzkopf M, Fornace ME, Acharya A, Artavanis G, Stegmaier J, Cunha A, Pierce NA. Third-generation in situ hybridization chain reaction: multiplexed, quantitative, sensitive, versatile, robust. Development. 2018;145(12):165753. 10.1242/dev.165753 PMC6031405 29945988

[55] Ahuja N, Hwaun E, Pungor JR, Rafiq R, Nemes S, Sakmar T, Vogt MA, Grasse B, Quiroz JD, Montague TG, Null RW. Creation of an albino squid line by CRISPR-Cas9 and its application for in vivo functional imaging of neural activity. Current Biology. 2023;33(13):2774-83. 10.1016/j.cub.2023.05.066 PMC12628842 37343558

[56] Andéol Y. Early transcription in different animal species: implication for transition from maternal to zygotic control in development. Roux’s Archives Dev Biology. 1994;204:3–10. 10.1007/BF00189062 28305800

[57] Tadros W, Lipshitz HD. The maternal-to-zygotic transition: a play in two acts. Development. 2009;136(18):3033–42. 19700615 10.1242/dev.033183

[58] Forbes A, Lehmann R. Nanos and pumilio have critical roles in the development and function of drosophila germline stem cells. Development. 1998;125(4):679–90. 9435288 10.1242/dev.125.4.679

[59] Xu R, Li Q, Yu H, Kong L. Oocyte maturation and origin of the germline as revealed by the expression of Nanos-like in the Pacific oyster *Crassostrea gigas*. Gene. 2018;663:41–50. 29660519 10.1016/j.gene.2018.04.021

[60] Carlos S, Jutglar L, Borrell I, Hunt DF, Ausio J. Sequence and characterization of a sperm-specific histone H1-like protein of *Mytilus californianus*. J Biol Chem. 1993;268(1):185–94. 7677995

[61] Cragg SM. The biology of scallop larvae. Scallop: Biology, Ecology and Aquaculture. 1991:75–132.

[62] Arenas-Mena C, Wong KS, Arandi-Forosani N. Ciliary band gene expression patterns in the embryo and trochophore larva of an indirectly developing polychaete. Gene Expr Patterns. 2007;7(5):544–9. 17350349 10.1016/j.modgep.2007.01.007

[63] Paganos P, Voronov D, Musser JM, Arendt D, Arnone MI. Single-cell RNA sequencing of the *Strongylocentrotus purpuratus* larva reveals the blueprint of major cell types and nervous system of a non-chordate deuterostome. Elife. 2021;10:e70416. 34821556 10.7554/eLife.70416 PMC8683087

[64] Fan S, Zhou D, Xu Y, Yu D. Cloning and functional analysis of BMP3 in the Pearl oyster (*Pinctada fucata*). J Appl Anim Res. 2019;47(1):250–61.

[65] Perry KJ, Lyons DC, Truchado-Garcia M, Fischer AH, Helfrich LW, Johansson KB, Diamond JC, Grande C, Henry JQ. Deployment of regulatory genes during gastrulation and germ layer specification in a model spiralian mollusc crepidula. Dev Dyn. 2015;244(10):1215–48. 26197970 10.1002/dvdy.24308

[66] Le Gouar M, Lartillot N, Adoutte A, Vervoort M. The expression of a caudal homologue in a mollusc, *Patella vulgata*. Gene Expr Patterns. 2003;3(1):35–7. 12609599 10.1016/s1567-133x(02)00091-1

[67] Johnson AB, Lambert JD. The caudal parahox gene is required for hindgut development in the mollusc *Tritia* (aka *Ilyanassa*). Dev Biol. 2021;470:1–9. 33191200 10.1016/j.ydbio.2020.10.010

[68] Lartillot N, Le Gouar M, Adoutte A. Expression patterns of fork head and goosecoid homologues in the mollusc Patella vulgata supports the ancestry of the anterior mesendoderm across Bilateria. Development genes and evolution. 2002;212(11):551-61. 10.1007/s00427-002-0274-8 12459924

[69] Huan P, Liu G, Wang H, Liu B. Identification of a tyrosinase gene potentially involved in early larval shell biogenesis of the Pacific oyster *Crassostrea gigas*. Dev Genes Evol. 2013;223:389–94. 23897397 10.1007/s00427-013-0450-z

[70] Liu G, Huan P, Liu B. A SoxC gene related to larval shell development and co-expression analysis of different shell formation genes in early larvae of oyster. Dev Genes Evol. 2017;227:181–8. 28280925 10.1007/s00427-017-0579-2

[71] Min Y, Li Q, Yu H. Characterization of larval shell formation and CgPOU2F1, CgSox5, and CgPax6 gene expression during shell morphogenesis in *Crassostrea gigas*. Comp Biochem Physiol B: Biochem Mol Biol. 2023;263:110783. 35926704 10.1016/j.cbpb.2022.110783

[72] Salamanca-Díaz DA, Calcino AD, de Oliveira AL, Wanninger A. Non-collinear hox gene expression in bivalves and the evolution of morphological novelties in mollusks. Sci Rep. 2021;11(1):3575. 33574385 10.1038/s41598-021-82122-6 PMC7878502

[73] Marin F, Corstjens P, de Gaulejac B, de Vrind-De Jong E, Westbroek P. Mucins and molluscan calcification: molecular characterization of mucoperlin, a novel mucin-like protein from the nacreous shell layer of the fan mussel Pinna nobilis (Bivalvia, *Pteriomorphia*). J Biol Chem. 2000;275(27):20667–75. 10770949 10.1074/jbc.M003006200

[74] Yu H, Li H, Li Q. Molecular characterization and expression profiles of myosin essential light chain gene in the Pacific oyster *Crassostrea gigas*. Comp Biochem Physiol B: Biochem Mol Biol. 2017;213:1–7. 28735975 10.1016/j.cbpb.2017.07.007

[75] Gulati GS, Sikandar SS, Wesche DJ, Manjunath A, Bharadwaj A, Berger MJ, Ilagan F, Kuo AH, Hsieh RW, Cai S, Zabala M. Single-cell transcriptional diversity is a hallmark of developmental potential. Science. 2020;367(6476):405–11. 31974247 10.1126/science.aax0249 PMC7694873

[76] Knirr S, Azpiazu N, Frasch M. The role of the NK-homeobox gene slouch (S59) in somatic muscle patterning. Development. 1999;126(20):4525–35. 10498687 10.1242/dev.126.20.4525

[77] Doe CQ, Chu-LaGraff Q, Wright DM, Scott MP. The prospero gene specifies cell fates in the *drosophila* central nervous system. Cell. 1991;65(3):451–64. 1673362 10.1016/0092-8674(91)90463-9

[78] Zeidler MP, Perrimon N, Strutt DI. The four-jointed gene is required in the Drosophila eye for ommatidial polarity specification. Curr Biol. 1999;9(23):1363–72. 10607560 10.1016/s0960-9822(00)80081-0

[79] Popov VM, Wu K, Zhou J, Powell MJ, Mardon G, Wang C, et al. The dachshund gene in development and hormone-responsive tumorigenesis. Trends Endocrinol Metab. 2010;21(1):41–9. 19896866 10.1016/j.tem.2009.08.002 PMC2818438

[80] Yurchenko OV, Skiteva OI, Voronezhskaya EE, Dyachuk VA. Nervous system development in the Pacific Ocean oyster, *Crassostrea gigas* (Mollusca: Bivalvia). Front Zool. 2018;15:1–21. 29681988 10.1186/s12983-018-0259-8 PMC5896133

[81] Nederbragt AJ, te Welscher P, van den Driesche S, van Loon AE, Dictus WJ. Novel and conserved roles for orthodenticle/otx and orthopedia/otp orthologs in the gastropod mollusc *Patella vulgata*. Dev Genes Evol. 2002;212:330–7. 12185486 10.1007/s00427-002-0246-z

[82] Latham KE, Schultz RM. Embryonic genome activation. Front Biosci. 2001;6(D748–D759):10–2741. 10.2741/latham 11401780

[83] Graf A, Krebs S, Zakhartchenko V, Schwalb B, Blum H, Wolf E. Fine mapping of genome activation in bovine embryos by RNA sequencing. Proc Natl Acad Sci. 2014;111(11):4139–44. 24591639 10.1073/pnas.1321569111 PMC3964062

[84] Pérez-Montero S, Carbonell A, Azorín F. Germline-specific H1 variants: the sexy linker histones. Chromosoma. 2016;125(1):1–3. 25921218 10.1007/s00412-015-0517-x

[85] Doniach T, Hodgkin J. A sex-determining gene, fem-1, required for both male and hermaphrodite development in *Caenorhabditis elegans*. Dev Biol. 1984;106(1):223–35. 6541600 10.1016/0012-1606(84)90077-0

[86] Galindo-Torres P, Ventura-López C, Llera-Herrera R, Ibarra AM. A natural antisense transcript of the *fem-1* gene was found expressed in female gonads during the characterization, expression profile, and cellular localization of the *fem-1* gene in Pacific white shrimp *Penaeus vannamei*. Gene. 2019;706:19–31. 31028869 10.1016/j.gene.2019.04.066

[87] Teaniniuraitemoana V, Huvet A, Levy P, Klopp C, Lhuillier E, Gaertner-Mazouni N, et al. Gonad transcriptome analysis of pearl oyster *Pinctada margaritifera*: identification of potential sex differentiation and sex determining genes. BMC Genomics. 2014;15:1–20. 24942841 10.1186/1471-2164-15-491 PMC4082630

[88] Bickel JS, Chen L, Hayward J, Yeap SL, Alkers AE, Chan RC. Structural maintenance of chromosomes (SMC) proteins promote homolog-independent recombination repair in meiosis crucial for germ cell genomic stability. PLoS Genet. 2010;6(7):e1001028. 20661436 10.1371/journal.pgen.1001028 PMC2908675

[89] Tong Y, Zhang Y, Huang J, Xiao S, Zhang Y, Li J, et al. Transcriptomics analysis of *Crassostrea hongkongensis* for the discovery of reproduction-related genes. PLoS ONE. 2015;10(8):e0134280. 26258576 10.1371/journal.pone.0134280 PMC4530894

[90] Ziegenhain C, Vieth B, Parekh S, Reinius B, Guillaumet-Adkins A, Smets M, Leonhardt H, Heyn H, Hellmann I, Enard W. Comparative analysis of single-cell RNA sequencing methods. Mol Cell. 2017;65(4):631–43. 28212749 10.1016/j.molcel.2017.01.023

[91] Extavour CG, Akam M. Mechanisms of germ cell specification across the metazoans: epigenesis and preformation. Development. 2003;130(24):5869–84. 14597570 10.1242/dev.00804

[92] Obata M, Komaru A. The mechanisms of primordial germ cell determination during embryogenesis in molluscan species. Invertebr Surv J. 2012;9(2):223–9.

[93] Hejnol A, Martindale MQ. Acoel development indicates the independent evolution of the bilaterian mouth and anus. Nature. 2008;456(7220):382–6. 18806777 10.1038/nature07309

[94] Lyons DC, Perry KJ, Henry JQ. Spiralian gastrulation: germ layer formation, morphogenesis, and fate of the blastopore in the slipper snail *Crepidula fornicata*. EvoDevo. 2015;6:1–34. 26664718 10.1186/s13227-015-0019-1 PMC4673862

[95] Schulreich SM, Salamanca-Díaz DA, Zieger E, Calcino AD, Wanninger A. A mosaic of conserved and novel modes of gene expression and morphogenesis in mesoderm and muscle formation of a larval bivalve. Organisms Divers Evol. 2022;22(4):893–913. 10.1007/s13127-022-00569-5 PMC9649484 36398106

[96] Lartillot N, Lespinet O, Vervoort M, Adoutte A. Expression pattern of brachyury in the mollusc *Patella vulgata* suggests a conserved role in the establishment of the AP axis in bilateria. Development. 2002. 10.1242/dev.129.6.1411. 11880350 10.1242/dev.129.6.1411

[97] Jackson DJ, Degnan BM. The importance of evo-devo to an integrated understanding of molluscan biomineralisation. J Struct Biol. 2016;196(2):67–74. 26792641 10.1016/j.jsb.2016.01.005

[98] Sachslehner A, Zieger E, Calcino A, Wanninger A. Hes and mox genes are expressed during early mesoderm formation in a mollusk with putative ancestral features. Sci Rep. 2021;11(1):18030. 34504115 10.1038/s41598-021-96711-y PMC8429573

[99] Lopez-Anido RN, Batzel GO, Ramirez G, Wang Y, Neal S, Lesoway MP, et al. The adult shell matrix protein repertoire of the marine snail crepidula is dominated by conserved genes that are also expressed in larvae. BMC Ecol Evol. 2024;24(1):120. 39277725 10.1186/s12862-024-02237-y PMC11401363

[100] Suzuki M, Nagasawa H. Mollusk shell structures and their formation mechanism. Can J Zool. 2013;91(6):349–66.

[101] Kniprath E. Ontogeny of the molluscan shell field: a review. Zool Scr. 1981;10(1):61–79.

[102] Eyster LS, Morse MP. Early shell formation during molluscan embryogenesis, with new studies on the surf clam, *Spisula solidissima*. Am Zool. 1984;24(4):871–82.

[103] Moor B. In: Verdonk NH, Van Den Biggelaar JAM, Tompa AS, editors. The mollusca: development. Volume 3. New York: Academic; 1983.

[104] Huang J, Zhang R. The mineralization of molluscan shells: some unsolved problems and special considerations. Front Mar Sci. 2022;9:874534.

[105] Liu G, Huan P, Liu B. Identification of three cell populations from the shell gland of a bivalve mollusc. Dev Genes Evol. 2020;230(1):39–45. 31960123 10.1007/s00427-020-00646-9

[106] Marie B, Joubert C, Tayalé A, Zanella-Cléon I, Belliard C, Piquemal D, et al. Different secretory repertoires control the biomineralization processes of prism and nacre deposition of the pearl oyster shell. Proc Natl Acad Sci U S A. 2012;109(51):20986–91. 23213212 10.1073/pnas.1210552109 PMC3529032

[107] Jackson HJ, Larsson J, Davison A. Quantitative measures and 3D shell models reveal interactions between bands and their position on growing snail shells. Ecol Evol. 2021;11(11):6634–48. 34141246 10.1002/ece3.7517 PMC8207382

[108] Parker LM, Ross PM, O’Connor WA, Pörtner HO, Scanes E, Wright JM. Predicting the response of molluscs to the impact of Ocean acidification. Biology. 2013;2(2):651–92. 24832802 10.3390/biology2020651 PMC3960890

[109] De Wit P, Durland E, Ventura A, Langdon CJ. Gene expression correlated with delay in shell formation in larval Pacific oysters (*Crassostrea gigas*) exposed to experimental ocean acidification provides insights into shell formation mechanisms. BMC Genomics. 2018;19:1–5. 29471790 10.1186/s12864-018-4519-y PMC5824581

[110] Liu Z, Zhang Y, Zhou Z, Zong Y, Zheng Y, Liu C, Kong N, Gao Q, Wang L, Song L. Metabolomic and transcriptomic profiling reveals the alteration of energy metabolism in oyster larvae during initial shell formation and under experimental ocean acidification. Sci Rep. 2020;10(1):6111. 32273532 10.1038/s41598-020-62963-3 PMC7145846

[111] Yurchenko OV, Savelieva AV, Kolotuchina NK, Voronezhskaya EE, Dyachuk VA. Peripheral sensory neurons govern development of the nervous system in bivalve larvae. EvoDevo. 2019;10:1–9. 31528326 10.1186/s13227-019-0133-6 PMC6743156

[112] Voronezhskaya EE, Khabarova MY, Nezlin LP. Apical sensory neurones mediate developmental retardation induced by conspecific environmental stimuli in freshwater pulmonate snails. Development. 2004. 10.1242/dev.01237. 15229179 10.1242/dev.01237

[113] Redl E, Scherholz M, Wollesen T, Todt C, Wanninger A. Expression of six3 and Otx in *Solenogastres* (Mollusca) supports an ancestral role in bilaterian anterior-posterior axis patterning. Evol Dev. 2018;20(1):17–28. 29243871 10.1111/ede.12245 PMC5814893

[114] Sun J, Zhang C, Gao F, Stathopoulos A. Single-cell transcriptomics illuminates regulatory steps driving anterior-posterior patterning of drosophila embryonic mesoderm. Cell Rep. 2023. 10.1016/j.celrep.2023.113289. 37858470 10.1016/j.celrep.2023.113289 PMC10704487

