# Supplementary material for: Primordial germ cell specification and early developmental cell states in Pacific oyster
Source: BMC Genomics. 2025 Oct 23;26:951. doi: 10.1186/s12864-025-12122-7 (PMC12551290; doi:10.1186/s12864-025-12122-7)
Supplement: Supplementary file 2 — Supplementary Figures [file 12864_2025_12122_MOESM2_ESM.pdf]

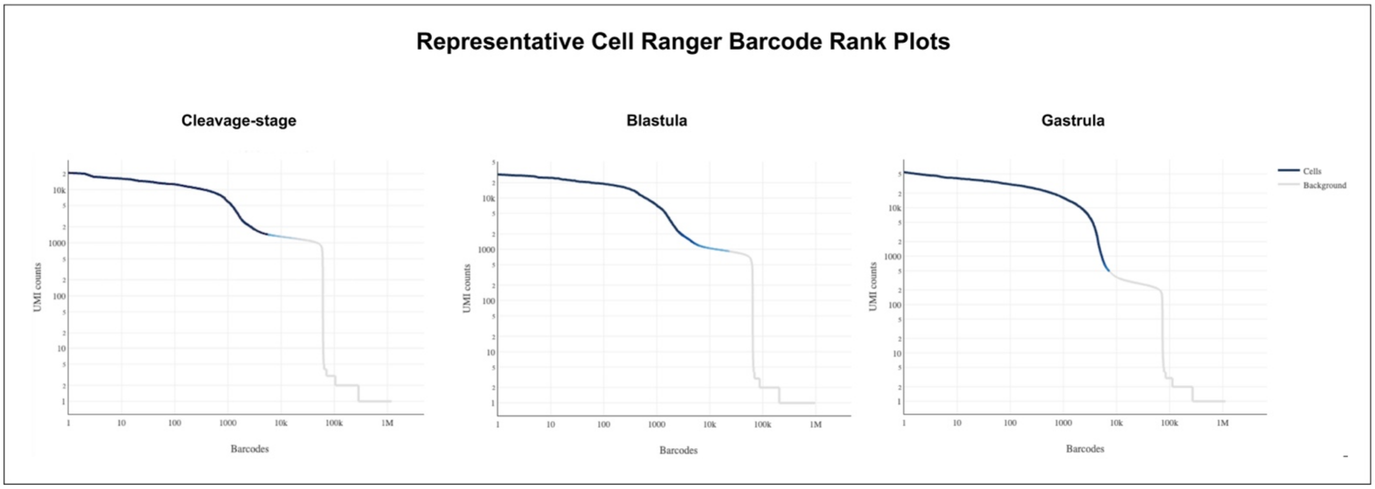

Figure S1. Representative Barcode Rank Plots

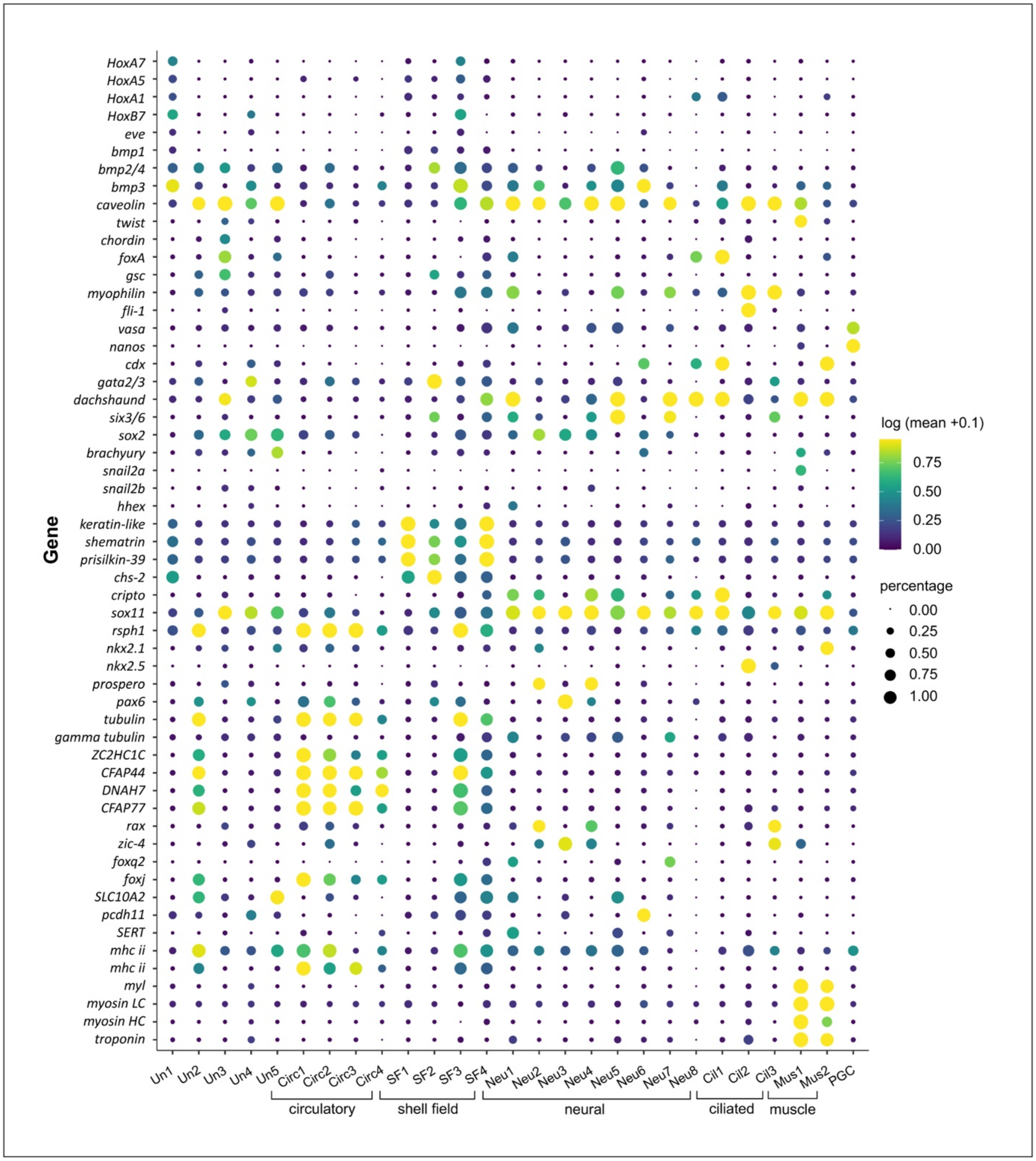

Figure S2. Dot plot illustrating expression of select gastrulae cluster identification genes from Table 1. Dot color represents expression level – maximum expression is set to 0.4 (i.e. all expression values ≥ 0.4 are yellow). Dot size represents fractional representation of cells expressing a gene.

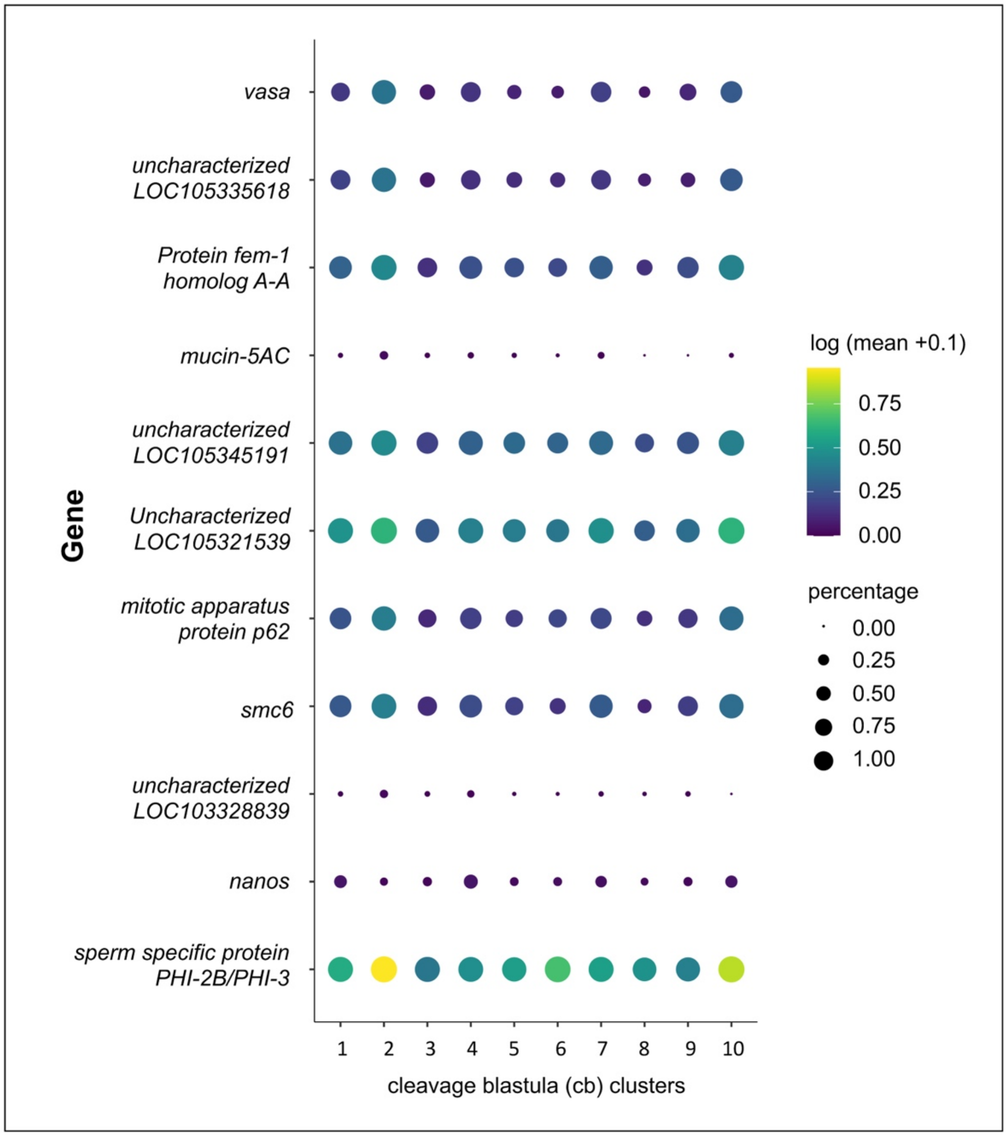

Figure S3. Genes associated with PGCs in oyster gastrulae are not specifically expressed in cleavage + blastula clusters. Dot plot illustrating expression of *vasa* and the top 10 PGC cluster marker genes in cleavage + blastula clusters. Dot color represents expression level and dot size represents fractional representation of cells expressing a gene.

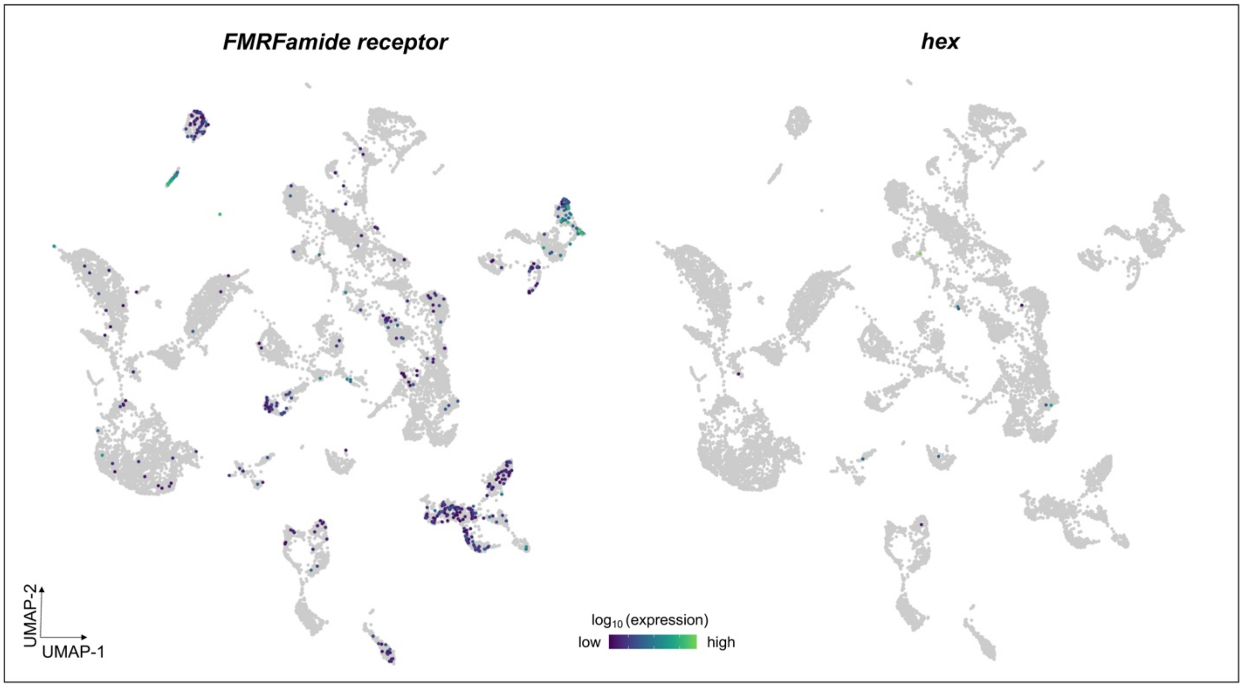

Figure S4. UMAP of neural markers not expressed at the gastrula stage in *C. gigas.* Expression patterns of select neural genes in UMAP space.

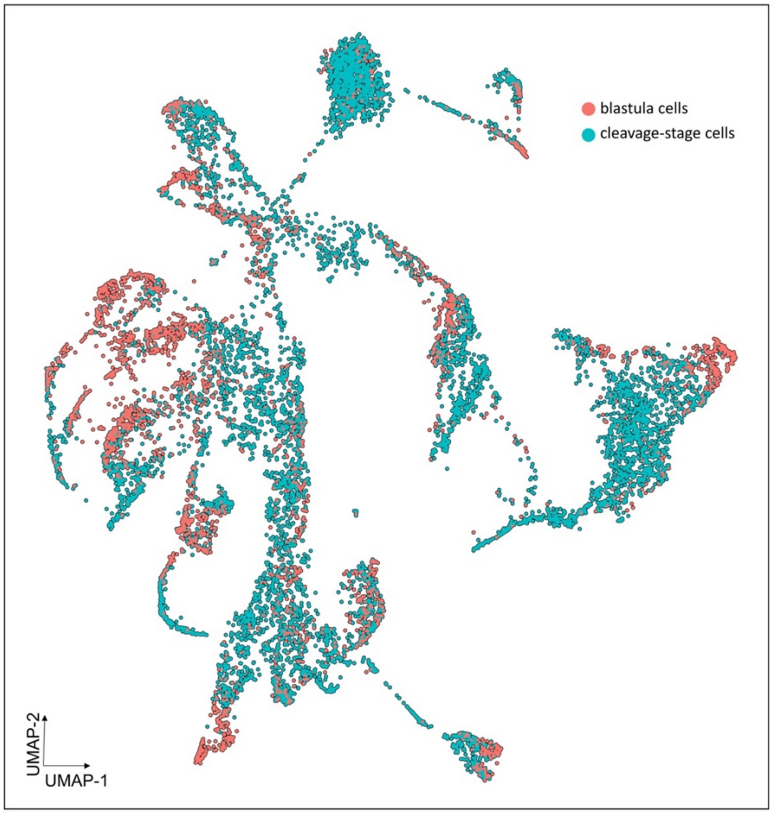

Figure S5. UMAP of cleavage + blastula cells color-coded according to library type. Colors: pooled cleavage stages (CP) and blastula (Bla).

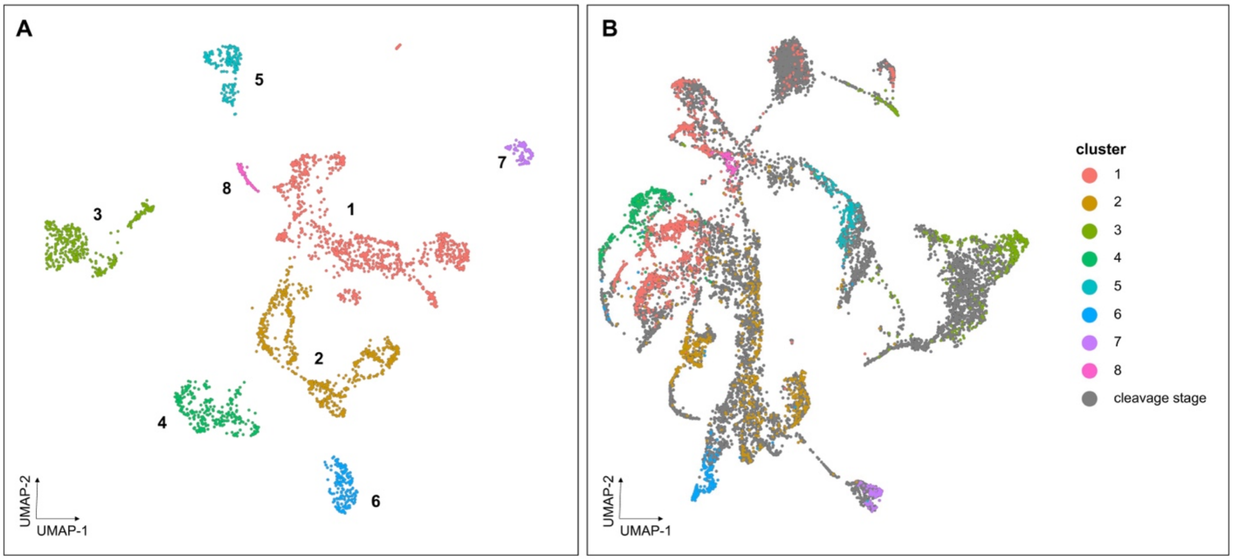

Figure S6. Cluster analysis of blastula cells visualized via UMAP. (A) UMAP plot in which cells are colored according to blastula clusters. (B) UMAP plot of cleavage-blastula cells, where cells are colored according to the blastula clusters and cells from the cleavage-stage embryos are gray.
